# Antimicrobial resistance profiling in poultry industry: a culture-independent resistome analysis and risk factor assessment

**DOI:** 10.1186/s12917-026-05334-w

**Published:** 2026-03-14

**Authors:** Sabah Ali, Mariam Hassan, Tamer Essam, Shaymaa Abdelmalek, Khaled F. Al-Amry

**Affiliations:** 1 Department of Microbiology, Faculty of Veterinary Medicine, Cairo University, Giza, 12211 Egypt; 2 Department of Microbiology and Immunology, Faculty of Pharmacy, Cairo University, Cairo, 11562 Egypt; 3 Department of Microbiology and Immunology, Faculty of Pharmacy, Galala University, New Galala City, Suez, Egypt

**Keywords:** Antibiotics, Antimicrobial resistance genes (ARGs), Broiler, Layers, Metagenomic, Molecular surveillance, One Health, PCR, Silent pandemic

## Abstract

Antimicrobial resistance (AMR) is a global health crisis, often termed as the “silent pandemic”. As emphasized by the One Health framework, the food production chain, especially poultry, serves as a critical point for monitoring AMR emergence and spread. In this study, we conducted a culture-independent screening of 27 antimicrobial resistance genes (ARGs) relevant to both human and veterinary health using cloacal samples collected from 9 farms across two of Egypt’s major poultry-producing cities. Alongside ARG screening, farm-level risk and managemental factors were assessed. Results showed that broiler farms had notably higher antimicrobial usage (AMU) and ARG prevalence compared to layer farms. Genes such as *sul*1 and *sul*2, *tet*A and *te*tM were significantly more abundant in broilers (*P* < 0.05, Fisher's Exact Test). Beta-lactam and sulphonamide resistance genes were widespread in both production systems. Key risk factors associated with increased ARG richness included bird type, lower farm biosecurity scores, summer season, diseased birds, use of multiple antibiotic classes and absence of veterinary prescriptions. Cluster analysis revealed *parC*, *aac* (3*)-*1a, *sul*2, *bla*TEM, *intI*1, *tet*M, *tet*A, and *sul*1 as the most prevalent genes among the screened samples. Additionally, *intI*1, a known marker of horizontal transfer, was detected in 79% of the samples and significantly co-occurred with tetracycline, beta-lactam, sulphonamide, and quinolone resistance genes (*P* >0.05, Fisher's Exact Test). To the best of our knowledge, this is the first study in Kalyoubia and Giza, two of Egypt’s major poultry trading hubs to comprehensively assess ARGs using a rapid, culture-independent PCR-based resistome analysis, highlighting critical AMR risks and its relation to ARGs’ richness in these poultry sectors.

**Supplementary Information:**

The online version contains supplementary material available at 10.1186/s12917-026-05334-w.

## Introduction

Antimicrobial resistance (AMR) is a global public health concern, directly responsible for more than 700,000 deaths annually, and reaching an estimated death toll of 10 million deaths per year by 2050 [1, 2]. Another significant factor that worsens the AMR scenario is the excessive overuse of antibiotics across human, animal, and environmental sectors, along with the transfer of antibiotic-resistant bacteria (ARB) and antimicrobial-resistant genes (ARG) between these highly interlinked and interdependent sectors, making antimicrobial resistance a more complex and challenging issue that must be addressed under a One Health perspective [3]. In this regard, the World Health Organization’s global action plan proposed several international and national strategies to tackle the AMR crises and to adopt a collective One Health approach, with food chain surveillance highlighted as a key starting point [4, 5].

Poultry farming plays a crucial role in the food chain, serving as one of the most popular sources of protein for millions of people worldwide, especially in low and middle-income countries (LMICs) [6, 7]. In Egypt, the poultry industry has rapidly expanded to meet the growing consumer demand for affordable animal protein. By 2018, the commercial broiler sector alone produced 690 million chickens, gaining approximately 46.8 billion EGP (Egyptian Pound) from poultry meat [4]. As a result, large quantities of antimicrobials are frequently used across Egyptian poultry farms to prevent and treat diseases, enhance animal productivity, boost immunity, and improve egg production and growth rates in poultry flocks [8, 9]. The excessive use, misuse, and abuse of antimicrobials without proper regulation and prescription within these farms have become major drivers for AMR development [10]. Accordingly, it is crucial for the Egyptian human and veterinary public health sectors to develop targeted interventions to address AMR challenges through continuous monitoring and surveillance of AMR and its associated risk factors in poultry farms [11]. This can be implemented through monitoring antimicrobial use and conducting screenings for antimicrobial-resistance genes and antibiotic-resistant bacteria at farm levels [12] enhancing biosecurity measures, and ensuring proper hygiene and managemental practices [13].

In this direction, numerous attempts and studies in Egypt have focused on screening AMR pathogens associated with poultry, such as *Escherichia coli*, *Salmonella serovars*, *Campylobacter spp.*, *Enterobacter spp.* and *Klebsiella spp*., using traditional phenotypic and culturable methods [14, 15]. However, these culture-based methods only capture a small fraction of the microbial diversity in the environment, leaving many potential sources of resistance unexamined [16]. Antibiotic-resistant bacteria, whether pathogenic, commensal, culturable, or unculturable, can transfer antimicrobial resistance genes and mobile genetic elements (MGE), such as plasmids, integrons, and transposons, within the environment via horizontal gene transfer (HGT). Additionally, free ARGs can be transformed into bacteria, leading to the transmission of untreatable diseases with high morbidities and mortalities, posing a major risk to public health [17].

Given these challenges, there is a growing need for more comprehensive surveillance techniques. Culture-independent methods, such as PCR-based techniques, quantitative PCR (qPCR), and metagenomics, offer more accurate and inclusive approaches to detect the full spectrum of antimicrobial resistance genes in the environment [18]. Metagenomics provides a non-targeted, unbiased method that offers extensive information on the resistance patterns harbored by all bacteria (i.e., the resistome) in the investigated samples, rather than only in the selected colonies [19, 20]. However, it is more costly and complex. In contrast, PCR-based methods are more targeted, practical, and cost-effective, as they screen specific genes with known nucleotide sequences using designed primers [18, 21]. These methods are also rapid and ideal for large-scale AMR screening, particularly in LMICs, where cost remains a primary barrier in research.

This study aimed to address the gaps in the current surveillance practices by using PCR-based methods to (1) investigate the most common ARGs in two high-production governorates, Kalyoubia and Giza, in Egypt. Additionally, this study (2) explored the managemental risk factors associated with the presence of ARGs in poultry farms. To the best of our knowledge, our study is the first to screen ARGs belonging to 7 widely used antimicrobial agents in veterinary and human medicine and 1 class of mobile gene (integrase) simultaneously in correlation with its related risk factors using a cheap practical PCR-based resistome analysis.

## Materials and methods

Unless otherwise specified, all experiments were conducted under aseptic conditions.

### Study area

**Fig. 1:**
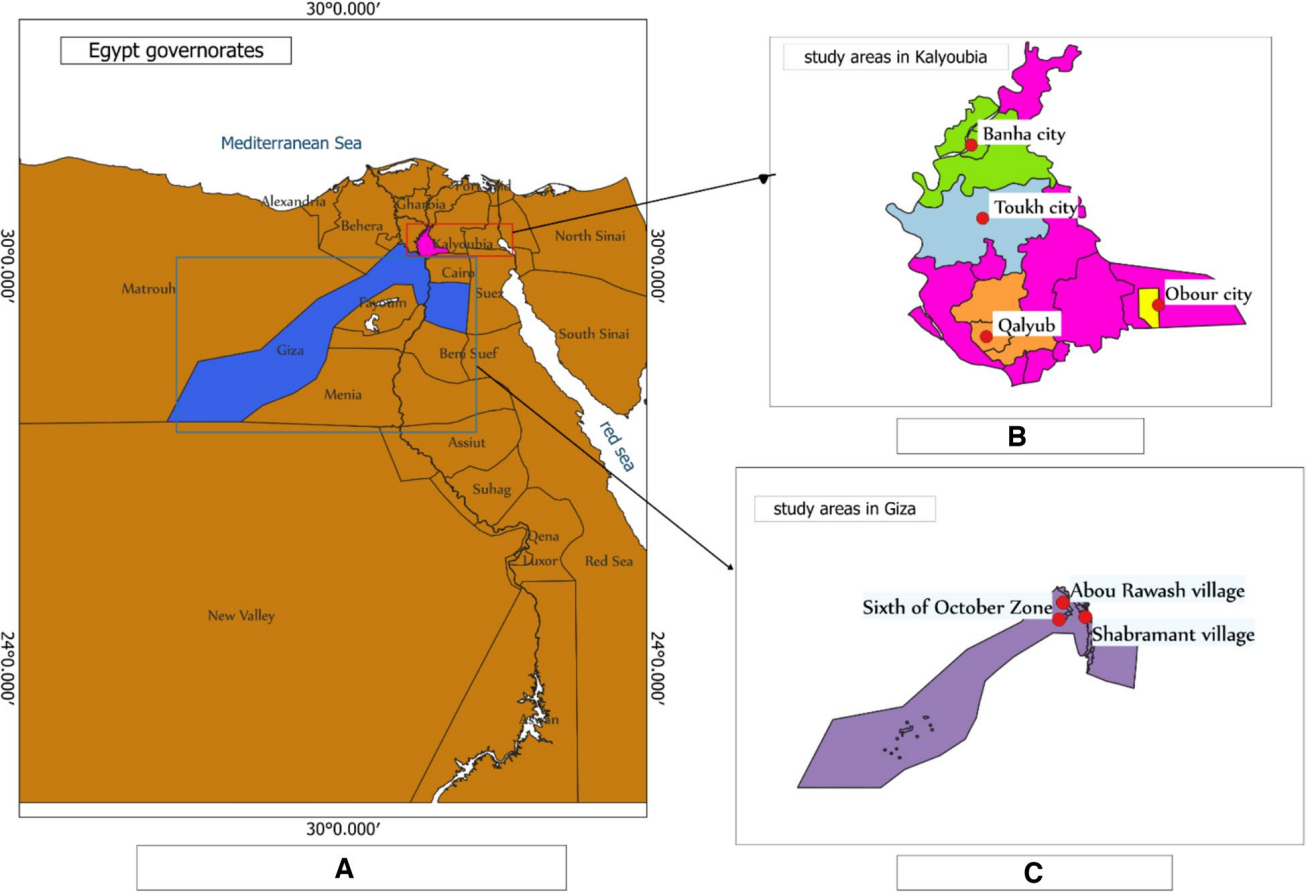
Geographical map showing studied governorates and districts in Egypt. **A** Map shows Kalyoubia and Giza governates (2 high poultry producing area) chosen for our study. **B** Map shows the selected districts within Kalyoubia. **C** Map shows the selected areas within Giza. (Designed by using QGIS v 3.40.3 software)

This study was carried out between October 2023 and August 2024 in two of the most important governorates for poultry production in Egypt, Kalyoubia and Giza (Fig. 1A). Kalyoubia and Giza are both part of the Greater Cairo metropolitan area and border the capital city, Cairo from the north and the south of the Nile River, respectively. These two governorates are dominated by a warm, Mediterranean climate on the northern coast, which is characterized by hot arid summers (around 30 °C high), mild winters (around 18/19 °C high and around 9/10 °C low), and high humidity in the Nile Delta region. Collectively, with their strategic proximity to Cairo and its high poultry demand, along with a favorable climate and fertile land, Kalyoubia and Giza have become major trading hubs for poultry and poultry products. Kalyoubia alone produces an annual average of 50.5 million broilers and 362.9 million eggs, while Giza poultry farms produce an annual average of 41.4 million broilers and 1.069 billion eggs [4]. Hence, these two provinces were selected as study areas for our study.

### Study design and selection criteria

A cross-sectional survey study was conducted to investigate the prevalence of antimicrobial resistance genes (ARG) in poultry farms and its associated risk factors in seven districts of high poultry production within the preselected provinces. Four districts were located in Kalyoubia (Obour City, Banha, Toukh, and Qalyub), and three of them were in Giza Governorate (Shabramant, Abu Rawash, and Sixth of October City), as illustrated in Fig. 1 (B and C). A total of nine poultry farms were randomly recruited, including five broiler (56%) and four-layer farms (44%). From these farms, 220 samples (44 pooled samples) were collected; broilers (55%, *n* = 120) and layers (45%,* n* = 100) as shown in Table 1. The enrollment criteria included farms with all-in all-out production systems. Eligible farms included medium (500-5000birds) to large-scale commercial & industrial (5,001–100,000 birds) farms. The enrolled study birds involved both healthy and sick chickens. Enrolled study birds included both sexes and birds > 2 weeks of age. The exclusion criteria included small-scale poultry farms, as they often manage mixed flocks with little to no production records [22]. Additionally, birds less than two weeks old were also excluded for biosafety and infection risk concerns from farm breeders. The nine poultry farms included in our study were randomly recruited from the population of eligible farms in the study area, based on their willingness to participate and accessibility. The geographic illustration was created using QGIS v 3.40.3 software (https://www.qgis) (Fig. 1).

**Table 1:** Cloacal samples collected from Egyptian poultry farms

Farm ID	Farm Category	Distribution		Location (District)	Governorate
		**No. of samples (N)**	**No. of pools (n)**		
FB1	Broiler	20	4	Qalub	Kalyoubia
FB2		25	5	Toukh	Kalyoubia
FB3		20	4	Obour City	Kalyoubia
FB4		20	4	Shabramant village	Giza
FB5		35	7	Abou Rawash	Giza
FL1	Layer	30	6	Banha	Kalyoubia
FL2		20	4	Obour city	Kalyoubia
FL3		30	6	Sixth of October City	Giza
FL4		20	4	Sixth of October City	Giza
Total farms	Total categories	Total No. of samples (N)	Total No. of pools (n)	Total districts	Total governorates
9	2	220	44	7	2

### Questionnaire

A brief questionnaire with 26 questions was constructed and divided into 5 key sectors. The first sector covered general sampling questions (location, code, type of samples, date/season, and number of samples) and the bird’s demographic data (status, poultry farm, breed & age). The second sector focused on the animal’s case history (disease diagnosis and mortality rates). The third sector addressed the farm characteristics (flock size and housing type). The fourth and the longest sector detailed the biosafety practices used in the farm, using 10 points. Farms that scored 5 out of 10 points or lower had low biosecurity, while those that scored 6 to 10 points were considered of high biosecurity [13, 23]. Finally, the last sector included data on antibiotics use (generic name of antibiotics given, and other medications used). The questionnaire was translated to Arabic and answered by the owners or the veterinarian in charge of each farm during personal interviews while collecting the samples. The full questionnaire is available in Supplementary File SF1.

### Sample collection and preparation

Each farm was visited once for inspection, sampling and data collection. Samples were taken either on-site or at nearby poultry labs by the research investigator or by the veterinarian in charge. All sick and dead birds brought to the labs were sampled, while healthy birds were randomly selected in the farms and their numbers were predetermined by the farm manager.

A total of 220 cloacal swabs were collected from 9 farms: 120 from 5 broiler farms and 100 from 4-layer farms as mentioned in Table 1. Out of the nine farms, five (56%) were sampled in summer (four broiler farms and one layer farm), while the remaining four (44%) were sampled in winter (three-layer farms and one broiler farm). The distribution of samples across the farms and regions is shown in Table 1.

Before sampling, cloacal swabs were moistened with phosphate-buffered saline (PBS), and then after collection, the swab head was placed in a sterile container containing 1mL PBS, tightly sealed, and transported to the laboratory in an ice-box [24]. Upon arrival, the samples were vortexed well, and 200 μL PBS from each of 5 swabs, all originating from the same pen and status, were pooled together to form a single test sample. A total of 44 pooled samples (each representing 5 samples) were prepared and stored at −20°C for further investigation. All samples were collected according to standard operating procedures and OIE recommendations [25].

### Metagenomic DNA extraction

Metagenomic DNA was extracted from the 44 fecal pools using the QIAamp Fast DNA Stool Mini Kit (Qiagen, Cat No. 51604 Germany) as per the manufacturer’s instructions. Briefly, 200 μl of the pooled samples were added to 1 ml of InhibitEX buffer, vortexed for 1 min, heated at 70 °C for 5 min, and then centrifuged at full speed to pellet the stool particles. The supernatant was mixed with 200 μl of buffer AL and 15 μl of proteinase K, incubated at 70 °C for 10 min, and then 200 μl of absolute ethanol was added. After that, 600 μl the lysate was put onto a QIAamp spin column, centrifuged, and the filtrate was discarded. Buffer AW1 (500 μl) and AW2 (500 μl) were sequentially added, and the column was centrifuged to remove residual contaminants. After a final wash step, the DNA was eluted with double-distilled water (ddH_2_0). The eluate was stored at −20°C for long-term storage. DNA concentration and quality were determined using a nanophotometer (Nanophotometer P360, Implen) as shown in Supplementary Table ST1.

### Targeted resistome PCR-based screening for ARGs

#### Primers used and primer design

A total of 27 primer sets were used to detect clinically relevant ARGs and MGEs in poultry pooled fecal samples using qualitative PCR. These primers targeted seven major classes of antibiotic resistance and one mobile gene class (integron-integrase). The ARGs confer resistance to β-lactams (*n* = 10) [*bla*NDM, *bla*KPC, *bla*OXA-48, *bla*VIM, *bla*TEM, *bla*SHV, *bla*OXA, *bla*CTX-M, *bla*CMY-2, and *mec*A], quinolones (*n* = 4) [*qnr*A, *qnr*B, *qnr*S, and *par*C], macrolides (*n* = 2) [*erm*B & *msr*C], tetracyclines (*n* = 3) [*tet*A, *tet*B, and *tet*M], sulphonamides (*n* = 3) [*sul*1, *sul*2, and *sul*3], aminoglycosides *(n* = 2) [*aac*(3)-Ia, and *arm*A], and glycopeptides (*n* = 2) [*van*A and *van*B], all known as important antimicrobials used for poultry therapeutic purposes and/or for human medicine [21, 26]. Meanwhile, *intI*1 (class 1 integrase) was investigated as an important indicator of antimicrobial resistance gene dissemination [27]. New primers for Sulphonamide resistance genes (*sul*1, *sul*2, and *sul*3) were designed to improve sensitivity for these commonly persistent antimicrobials present in animal manure. Nucleotide sequences encoding sulphonamide resistance genes (*sul1*, *sul2*, and *sul3*) were downloaded from the GenBank Database (http://www.ncbi.nlm.nih.gov/), and aligned with the multiple-sequence alignment program CLUSTALX 2.0.11 [28]. Sequences were then aligned and compared with each other to create consensus sequences for the primer design templates using Invitrogen Oligo Perfect primer design software. The rest of the primers were selected from previously published reports. Table 2 demonstrates the primer sequences, annealing temperature, references, and amplicon length of all ARGs (*n* = 26) and MGEs (*n* = 1) used in this study. The sulfonamide primers used in this study were evaluated through several in-silico specificity screening tools such as Primer-BLAST (https://www.ncbi.nlm.nih.gov/tools/primer-blast), OligoEvaluator (https://www.oligoevaluator.com/) and Insilco-PCR amplication http://insilico.ehu.es/PCR/Amplify.php). Detailed scores and summary of designed primers evaluation is shown in Supplementary file 2.

**Table 2:** List of primers used for the detection of antibiotic resistance genes (ARGs)

Antimicrobial class	Gene name	Gene sequence (5–3) direction	PCR product size (bp)	Annealing temperature (°C)	reference
1. Beta-lactam a. Extended-spectrum beta-lactamase (ESBL) resistance genes	*bla*_TEM_	F: CGCCGCATACACTATTCTCAG AATGA R: ACGCTCACCGGCTCCAGATTTAT	445	62	[29]
	*bla*_SHV_	F: CTTTATCGGCCCTCACTCAA R: AGGTGCTCATCATGGGAAAG	237	62	
	*bla*_CTX-M_	F: ATGTGCAGYACCAGTAARGTKATGGC R: TGGGTRAARTARGTSACCAGAAYCAGCGG	593	62	
	*bla*_OXA-1_	F: ACACAATACATATCAACTTCGC R: AGTGTGTTTAGAATGGTGATC	813	62	
b. Carbapenem resistance genes	*bla*_KPC_	F: ATGTCACTGTATCGC CGTCT R: TTTTCAGAGCCTTACTGCCC	882	55	
	*bla*_OXA-48_	F: TTGGTGGCATCGATTATCGG R: GAGCACTTCTTTTGTGAT GGC	743	55	
	*bla*_NDM_	F: GGTTTGGCGATC TGG TTTTC R: CGGAATGGCTCATCACGATC	621	55	
	*bla*_VIM_	F: GGTCTCATTGTCCGTGATGGTGAT GAG R: CTCGATGAGAGTCCTTCTAGAG	261	55	
C. Amp C beta-lactamase resistance genes	*bla*_CMY-2_	F: AGCGATCCGGTCACGAAATA R: CCCGTTTTATG CACCCATGA	695	61	
D. Methicillin resistance genes	*mec*A	F: TGGCTCAGGTACTGCTATCCAC R: AGTTCTGCAGTACCGGATTTGC	776	60	[30]
2. Tetracycline resistance genes	*tet*A	F: GGCCTCAATTTCCTGACG R: AAGCAGGATGTAGCCTGTG	372	55*	[31]
	*tet*B	F: TTCGGCATTCTGAATCTCAC R: ATGATCTAACCCTCGGTCTC	634	60*	[32]
	*tet*M	F: ACAGAAAGCTTATTATATAAC R: TGGCGTGTCTATGATGTTCAC	171	47*	
3. Sulfonamide resistance genes	*sul*1	F: CGCACCGGAAACATCGCTGC R: TGAAGTTCCGCCGCAAGGCT	162	55	Designed in this study
	*sul*2	F: TCCGATGGAGGCCGGTATCTGG R: CGGGAATGCCATCTGCCTTGAG	190	68	Designed in this study
	*sul*3	F: AGTAGCTGCACCAATACGCT R: CAACTGAAGTGGGCGTTGTG	248	58	Designed in this study
4. Aminoglycoside resistance genes	*arm*A	F: AGGTTGTTTCCATTTCTGAG R: TCTCTTCCATTCCCTTCTCC	776	50*	[33]
	*aac* (3)-Ia	F: ATGGGCATC ATTCGCA R: TCTCGGCTTGAACGAATTGT	484	57*	[34]
5. Quinolones a. Quinolone resistance genes	*qnr*S	F: GACGTGCTAACTTGCGTGAT R: TGGCATTGTTGGAAACTTG	118	50*	[35]
	*qnr*A	F: TCAGCAAGAGGATTTCTCA R: GGCAGCACTATTACTCCCA	661	56*	[36]
	*qnr*B	F: TCCGCTGTCAGTTCTATGATCG R: TCCATGAGCAACGATGCCT	495	52*	[37]
b. Fluroquinolone resistance genes	*par*C	F: GCCTTGCGCTACATGAATTT R: ACCATCAACCAGCGGATAAC	287	47*	[38]
6. Glycopeptide (Vancomycin resistance genes)	*van*A	F: GGGAAAACGACAATTGC R: GTACAATGCGGCCGTTA	732	55*	[39]
	*van*B	F: AAGCTATGCAAGAAGCCATG R: CCGACAATCAAATCATCCTC	536	47*	
7. Macrolide resistance genes	*erm*B	F: GAAAAG GTACTCAACCAA ATA R: AGTAACGGTACTTAAATT GTT TAC	639	50	[40]
	*msr*C	F: AAGGAATCCTTCTCTCTCCG R: GTA AACAAA ATCGTTCCC G	342	55	
8. Mobile genetic element (class 1 integron integrase)	*intl*1	F: GGCTTCGTGATGCCTGCTT R: CATTCCTGGCCGTGGTTCT	146	57*	[41]

Asterisk (*) indicate that these temperatures were modified from the original reference to optimize amplification conditions in this study

#### PCR assay

The PCR reactions were performed in a thermocycler (SensoQuest, Germany) and 25 μl total volume, including 12.5 μl of 2X DreamTaq Green PCR Master Mix (Vilnius, Lithuania, Europe), 1 μl of each primer (10 mM), and water. The genes were grouped together according to their annealing temperature and band size for multiplex, duplex or uniplex PCR reactions. The PCR conditions and gene grouping are shown in Table 3. The PCR products were run on 1% agarose gel stained with ethidium bromide and visualized under UV transillumination.

**Table 3:** Grouping of antibiotic resistance genes (ARGs) into Uniplex, Duplex, and Multiplex PCR reactions with their respective amplification conditions

PCR reaction	Name of gene	PCR product size (bp)	Conditions	Type of PCR and annealing temperature
Gp 1	*bla* TEM	445 bp	Initial denaturation at 95°C/5min, Denaturation at 95°C/30s, Annealing at 62°C/90s, Extension at 72°C/60s, Final extension at 72°C/10min For 30 cycles	Multiplex (62 °C)
	*bla* SHV	237 bp		
	*bla* CTXM	593 bp		
	*bla* OXA	813 bp		
Gp 2	*bla* KPC	882 bp	Initial denaturation at 94°C/5min, Denaturation at 94°C/1min, Annealing at 55°C/1min, Extension at 72°C/1min, Final extension at 72°C/10min For 30 cycles	Multiplex (55 °C)
	*bla* OXA-48	743 bp		
	*bla* NDM	621 bp		
	*bla* VIM	261 bp		
Gp 3	*bla*CMY-2	695 bp	Initial denaturation at 94°C/5min, Denaturation at 94°C/1min, Annealing at 61°C/1min, Extension at 72°C/1min, Final extension at 72°C/10min For 30 cycles	Uniplex (61 °C)
Gp4	*mec*A	776bp	Initial denaturation at 95°C/5min, Denaturation at 95°C/45s, Annealing at 60°C/45s, Extension at 72°C/1.30min, Final extension at 72°C/10min For 30 cycles	Duplex (60 °C)
	*tet*B	634 bp		
Gp5	*sul*1	162 bp	Initial denaturation at 94°C/5min, Denaturation at 94°C/1min, Annealing at 55°C/1min, Extension at 72°C/1min, Final extension at 72°C/10min For 35 cycles	Duplex (55 °C)
	*tet*A	372 bp		
Gp6	*sul2*	190 bp	Initial denaturation at 95°C/5min, Denaturation at 95°C/30 s, Annealing at 68°C/45 s, Extension at 72°C/1min, Final extension at 72°C/7min For 30 cycles	Uniplex (68 °C)
Gp7	*sul3*	248 bp	Initial denaturation at 94°C/5min, Denaturation at 94°C/1min, Annealing at 58°C/1min, Extension at 72°C/1min, Final extension at 72°C/7min For 30 cycles	Uniplex (58 °C)
Gp8	*arm*A	776 bp	Initial denaturation at 94°C/5min, Denaturation at 94°C/1min, Annealing at 50°C/1min, Extension at 72°C/1min, Final extension at 72°C/7min For 35 cycles	Uniplex (50 °C)
Gp9	*aac* (3)-la	484 bp	Initial denaturation at 94°C/5min, Denaturation at 94°C/1min, Annealing at 57°C/1;15min, Extension at 72°C/1:30min, Final extension at 72°C/7min For 30 cycles	Duplex (57 °C)
	*intl*1	146 bp		
Gp10	*qnr*S	118 bp	Initial denaturation at 94°C/5min, Denaturation at 94°C/1min, Annealing at 50°C/1min, Extension at 72°C/1min, Final extension at 72°C/7min For 35 cycles	Uniplex (50 °C)
Gp11	*qnr*A	661 bp	Initial denaturation at 94°C/5min, Denaturation at 94°C/45 s, Annealing at 56°C/1min, Extension at 72°C/1.30min, Final extension at 72°C/10min For 35 cycles	Uniplex (56 °C)
Gp12	*qnr*B	495bp	Initial denaturation at 94°C/5min, Denaturation at 94°C/45 s, Annealing at 52°C/1min, Extension at 72°C/1.30min, Final extension at 72°C/10min For 35 cycles	Uniplex (52 °C)
Gp 13	*tet*M	171 bp	Initial denaturation at 95°C/5min, Denaturation at 95°C/45 s, Annealing at 47°C/1.30min, Extension at 72°C/1min, Final extension at 72°C/10min for 35 cycles	Triplex (47 °C)
	*par*C	287bp		
	*van*B	536bp		
Gp14	*van*A	732 bp	Initial denaturation at 95°C/5min, Denaturation at 95°C/45 s, Annealing at 55°C/1.30min, Extension at 72C/1min, Final extension at 72°C/10min For 35 cycles	Duplex (55 °C)
	*msr*C	342 bp		
Gp15	*erm*B	639 bp	Initial denaturation at 95°C/5min, Denaturation at 95°C/45 s, Annealing at 50C/1.30min, Extension at 72°C/1min, Final extension at 72°C/10min For 35 cycles	Uniplex (50° °C)

### Statistical analysis

Statistical analyses and graphical presentations were performed using GraphPad Prism® software (version 9.01). A p-value < 0.05 was considered statistically significant unless otherwise specified, and 95% confidence intervals were reported where appropriate. Data were summarized using descriptive statistics, including means ± standard deviations for continuous variables and frequencies and percentages for categorical variables. Differences in the prevalence of biosecurity practices between high and low biosecurity farms, and ARGs prevalence were assessed using Fisher’s exact test. Associations between biosecurity scores and prescription-based antibiotic use were evaluated using Pearson’s correlation coefficient. A clustered heatmap was generated to explore ARG patterns across samples using the *pheatmap* package in R software (version 4.4.3).

Because multiple pools were sampled from the same farms, observations were not independent. Therefore, univariate analyses (Fisher’s exact tests) were used only for exploratory, descriptive purposes. Variables showing apparent differences, along with biologically relevant factors, were included in a multivariable logistic regression to assess adjusted associations, with ARG richness categorized as high (≥ 8 ARGs) or low (< 8 ARGs). Multivariable analyses were conducted using R (version 4.4.3).

### Ethical approval

This study protocol was ethically approved by the Faculty of Veterinary Medicine, Cairo University, Egypt. All authors pledge that all procedures and ethical principles regarding the safety and rights of both healthy and diseased animals used in this survey met the regulations of the Committee of Ethics of Cairo University-Institutional Animal Care and Use (CU-IACUC), and were granted the study approval number: CU110520251157. Regarding animal participation, oral consent was obtained from poultry farm owners and breeders before sampling, and study codes were given to maintain owners’ confidentiality.

## Results

### Farm demographic characteristics and questionnaire data analysis

A total of 44 pooled samples were obtained from 9 poultry farms in two governorates: Giza (47.7%, *n* = 21) & Kalyoubia (52.2%, *n* = 23). These included 24 pools from broiler farms and 20 pools from layer farms as shown in Table 1. Among these samples, 20 (45.4%) samples were obtained from apparently healthy birds (mean mortality rate: 2 ± 1.31 birds/day) and 24 (54.5%) from diseased birds showing clinical signs (mean mortality rate: 11 ± 5.51 birds/day) (Supplementary Table ST2). The flock’s age ranged from 20 days to 35 weeks, with the majority being young chicks less than one month of age (47.7%, *n* = 21). Farms were categorized into 3 production systems [22, 42]. The vast majority of the farms belonged to medium scale production categories (44.4%, *n* = 18), rearing < 5000 birds per cycle, whereas large commercial (33.3%, *n* = 14) and intensive scale (22.2%, *n* = 12) were less frequent (Table 4). Seventy-seven percent (77.2%, *n* = 7/9) of the flocks were housed using deep litter management systems and only twenty-two percent (22.2%, *n* = 2/9) of the surveyed farms were cage-based. In broiler flocks, the predominant breed was Cobb (27.2%, *n* = 12), while Arbor Acres, Ross, and Baladi broilers were each represented at similar frequencies. In contrast, layer breeds were more evenly distributed, with Hy-line and Lohman each accounting for 13.6% (*n* = 6), and Baladi layer compromising 18.1% (*n* = 8) of the flock (Table 4).

**Table 4:** Risk factors and farm characteristics associated with ARG richness across surveyed poultry farms

Variable	Category	No. (%) of examined farms	No. (%) of examined pools	Average ARG richness ± SD	Statistics	
					***P* value (*P* < 0.05)**	**Test**
1. Region	Kalyoubia	5 (55.5)	23 (52.2)	7.87 ± 4.15	*P* = 0.9061	Two tailed t test
	Giza	4 (44.4)	21 (47.7)	7.71 ± 4.51		
2. Farm type	Broiler	5 (55.5)	24 (54.5)	9.5 ± 4.201	*P* = 0.0037*	Two tailed t test
	Layer	4 (44.4)	20 (45.4)	5.75 ± 3.477		
3. Breed of chicken	Ross	1 (11.1)	4 (9.1)	11.75 ± 5.85	*P* = 0.0009*	OWA, [TMT]
	Cobb	2 (22.2)	12 (27.2)	10.45 ± 3.37		
	Arbor	1 (11.1)	4 (9.1)	6.5 ± 5.27		
	Balidi broiler	1 (11.1)	4 (9.1)	7.5 ± 2.08		
	Hy-line	1 (11.1)	6 (13.6)	5.83 ± 1.72		
	Lohman white layer	1 (11.1)	6 (13.6)	9 ± 3.74		
	Baladi layer	2 (22.2)	8 (18.1)	3.25 ± 2.121		
4. Age of chicken	< 1Month	4 (44.4)	21 (47.7)	8.76 ± 4.93	*P* = 0.6185	OWA, [TMT]
	1–2 Month	3 (33.3)	13 (29.5)	7.46 ± 4.07		
	> 1 Month	2 (22.2)	10 (22.7)	6.8 ± 4.15		
5. Health status	Apparently healthy	4.5 (50)	20 (45.4)	5.1 ± 3.177	*P* < 0.0001*	Two tailed t test
	Diseased	4.5 (50)	24 (54.5)	10 ± 3.793		
6. Season	Summer	5 (55.5)	26 (59.1)	8.96 ± 3.783	*P* = 0.0180*	Two tailed t test
	Winter	4 (44.4)	18 (40.9)	6 ± 4.48		
7. Flock size	Medium scale (500–5000)	4 (44.4)	18 (40.9)	7.9 ± 4.646	*P* = 0.0079*	Two tailed t test
	Large commercial (> 5000–25,000)	3 (33.3)	14 (31.8)	5.3 ± 3.177		
	Large intensive (> 25,000)	2 (22.2)	12 (27.2)	10.4 ± 3.37		
8. Poultry housing system	Floor	7 (77.7)	32 (72.7)	7.9 ± 4.655	*P* = 0.7240	Two tailed t test
	Cages	2 (22.2)	12 (27.2)	7.4 ± 3.232		
9. Biosafety score	High	4 (44.4)	18 (40.9)	4.83 ± 3.13	*P* < 0.0001*	Two tailed t test
	Low	5 (55.5)	26 (59.1)	9.85 ± 3.77		
10. Antibiotic usage	With prescription	3 (33.3)	12 (27.2)	3.25 ± 2.12	*P* = 0.0013*	OWA, [TMT]
	Both	2 (22.2)	11 (25)	8.0 ± 3.07		
	Without prescription	4 (44.4)	21 (47.7)	9.38 ± 4.53		
11. Antibiotic classes	One class	4 (44.4)	13 (29.5)	9.54 ± 3.75	*P* = 0.0009*	OWA, [TMT]
	Two classes	2 (22.2)	18 (40.9)	5.06 ± 2.57		
	≥ Three classes	3 (33.4)	13 (29.5)	9.85 ± 4.81		

Total farms = 9, total pools = 44

*OWA* One Way ANOVA used to statistically compare 3 variable or more, via *TMT* Tukey multiple comparison test

Asterisk (*) indicates a significant correlation between the variable and the average ARG richness

### Farm biosecurity measures and antimicrobial agents’ usage

**Fig. 2:**
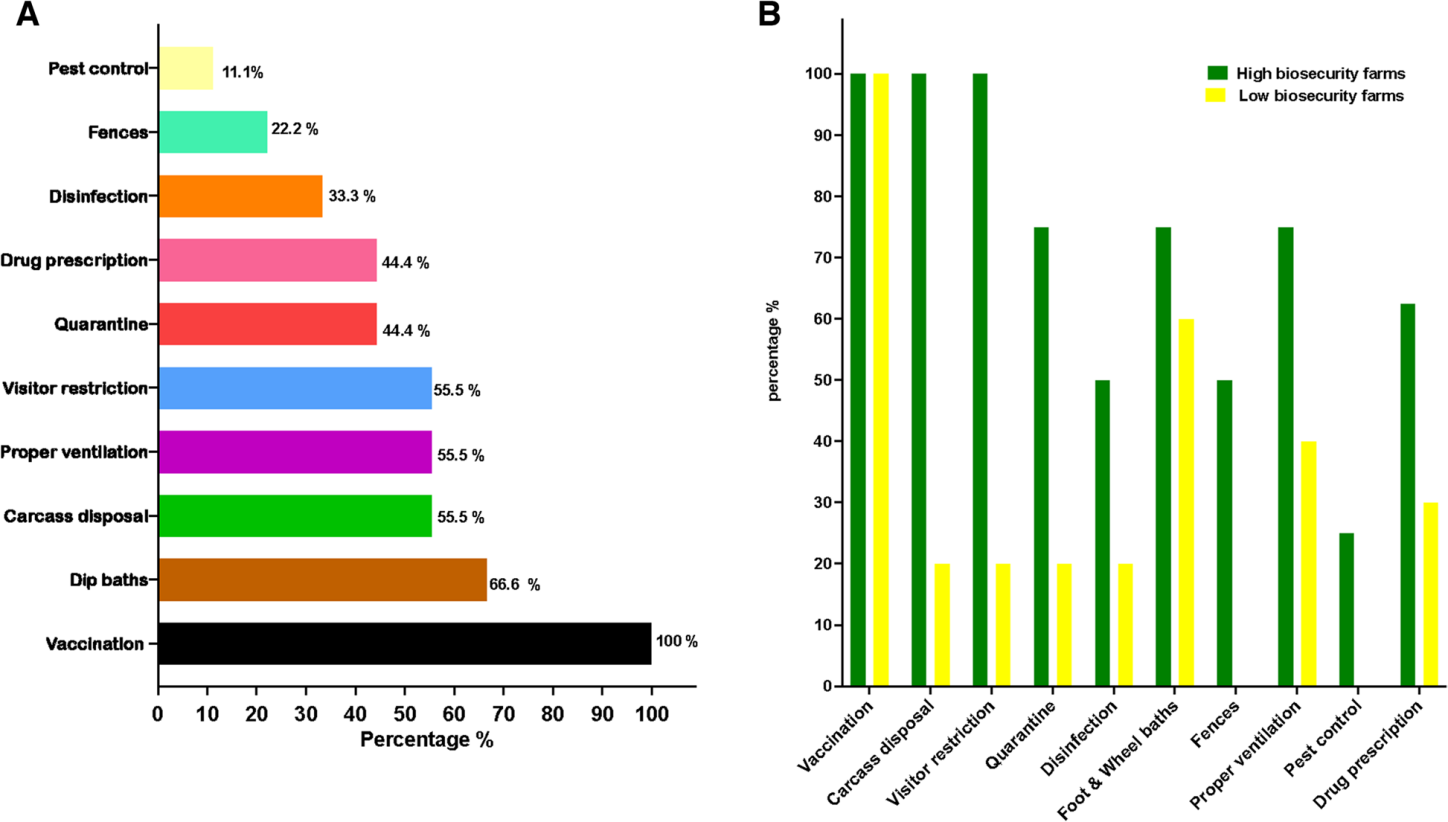
Biosecurity practices applied across surveyed farms.** A** Frequency of biosecurity practices across surveyed farms. **B** Percentages (%) of each biosecurity parameter in high biosecurity vs low biosecurity farms

All farms practiced biosecurity measures in varying degrees (Fig. 2A). Vaccination was exclusively used (100%, *n* = 9) in all farms, while pest control had the lowest implementation rate (11.1%, *n* = 1) across the farms. As for quarantine measures, they were implemented in 75% (*n* = 3/4) of high-biosecurity farms compared to 20% (*n* = 1/5) in low-biosecurity farms. Regular cleaning and disinfection of equipment, feeders and pens were reported in half of the high biosecurity farms (50%, *n* = 2/4) and one-fifth (20%,* n* = 1/5) of low biosecurity farms (Fig. 2B). Except vaccination (*p >* 0.05), all biosecurity practices, including supervised antibiotic usage, quarantine, proper carcass disposal, and other practices, were significantly more prevalent in high biosecurity farms compared to low biosecurity farms (Fisher's exact test, *P* < 0.05).

**Fig. 3:**
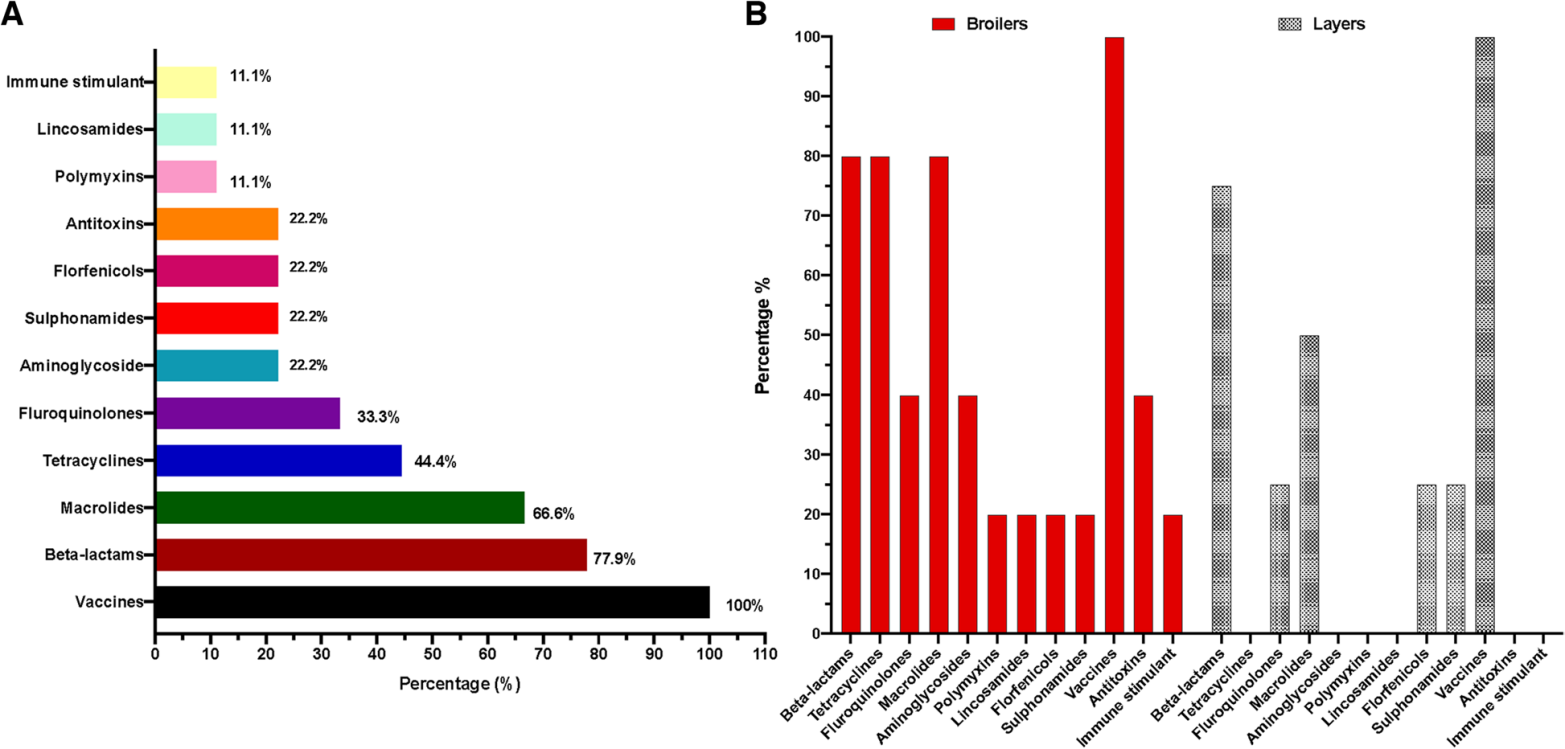
Medications applied across surveyed farms. **A** Frequency of medications and antimicrobial agents used across surveyed farms. **B** Antimicrobial usage and medications used in Broilers vs layers

Antimicrobial agents were used in all broiler and layer farms with notable differences. Beta-lactams were the most commonly used in both production types (Fig. 3A & B). Overall, layer farms had lower antimicrobial usage compared to broiler farms (Fig. 3B. In broiler farms, tetracyclines, macrolides, and beta-lactams were the most common antibiotics, with a prevalence rate of 80% (*n* = 4/5). However, beta-lactams (75%, *n* = 3/4), macrolides (50%, *n* = 2/4), and fluoroquinolones (25%, *n* = 1/4) were the most frequently used in layer farms, respectively. Detailed medication classes, generic names and their distribution across both production types are presented in Supplementary Table ST3. Alarmingly, only 33.3% (*n* = 3) of antimicrobial applications were based on veterinary prescriptions, indicating that the majority of farms applied antimicrobial agents without vet insight or with irregular prescriptions (Table 4). Furthermore, one-third (33.3%,* n* = 3) of farms reported using antibiotics from three or more distinct classes for treatment, prevention, or both, indicating widespread multidrug use (Table 4).

To explore the relationship between biosecurity practices and antimicrobial use, a Pearson correlation analysis was conducted. Statistical analysis revealed no significant correlation between biosecurity scores and prescription-based antibiotic use (r = 0.45, P = 0.22). Consequently, no association between these variables could be concluded.

### Overall prevalence and distribution of ARGs in poultry farms

**Fig. 4:**
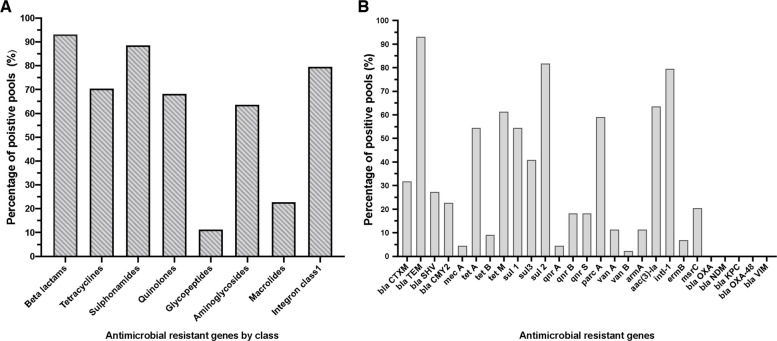
Relative abundance of ARGs and antimicrobial class across pooled samples.** A** Percent of pooled samples positive for eight antimicrobial classes resistance, total pooled samples *n* = 44, a class was considered positive if any or all genes within class was detected in the sample and marked (1); otherwise, if none was detected, it was marked (0). **B** Relative abundance of ARGs across screened pooled samples

All pooled samples were screened for ARGs conferring resistance to 7 antibiotic classes and one mobile gene class. All samples tested positive for at least one investigated ARG. A total of 343 amplicons were amplified across pooled samples, which belonged to 22 out of 27 targeted ARGs. (Supplementary Tables ST4 and ST5). The most frequently detected antimicrobial class was beta-lactams, with at least one gene identified in 93% (*n* = 41) of the samples. This was followed by sulphonamides (88%, *n* = 39) and tetracyclines (70%, *n* = 31) ARGs. In contrast, resistance genes related to macrolides and glycopeptides were detected in less than 25% of samples (Fig. 4A). To account for the potential bias introduced by unequal numbers of target genes per antibiotic class, prevalence was also examined at the individual-gene level. At the gene level, the top five predominant genes within screened samples were *bla*TEM (93%, *n* = 41), *sul*2 (82%, *n* = 36)*, intl* 1 (80%, *n* = 35), *aac* (3)-la (64%, *n* = 28), and *tet*M (61.3%, *n* = 27), corresponding to beta-lactams, sulphonamides, integrase/mobile elements, aminoglycosides, and tetracycline resistance, respectively. Whereas, the least frequent genes detected were *van*B (2%, *n* = 1), *qnr*A, and *mec*A (both 4.5%, *n* = 2). Notably, all four carbapenem-resistant genes (*bla*NDM, *bla*KPC, *bla*OXA-48, *bla*VIM) and one *bla*OXA-1 (ESBL-like variant), all belonging to beta-lactam resistance class, were found negative in all samples (Fig. 4B).

### Comparison of ARGs prevalence between broiler and layer flocks

In our study, ARGs were generally more prevalent in broiler than layer samples, except for β-lactams genes, where *bla*TEM was higher in layer (95%, *n* = 19/20) than broiler (91%, *n* = 22/24) samples, making β-lactams the most common AMR class in layers samples. Furthermore, the *arm*A gene was more frequent in layer samples (15%,* n* = 3/20) compared to broiler samples (8%, *n* = 2/24), although overall aminoglycoside resistance was higher in broiler samples. There was no significant difference in regard to the prevalence of the ARG classes between broiler and layer flocks except for tetracycline (*tet*A and *tet*M) and sulphonamide resistance genes (*sul*1 and *sul2*) (*P* < 0.05, Fisher's Exact Test). All broiler flocks (100%, *n* = 24) carried at least one sulphonamide-resistant gene, mainly *sul*2 (95%, *n* = 23) and *sul*1 (79%, *n* = 19) making sulphonamides the most common AMR class in broilers, followed by β-lactams and then tetracyclines. In layer samples, sulphonamides (75%, *n* = 15/20) ranked second after β-lactams (95%, *n* = 19/20), especially for *sul*2 and *sul*3, followed by aminoglycosides and quinolones, which were detected at similar rates (60%, *n* = 12/20) (Supplementary Figure SFig. 1 and SFig. 2). In particular, the top 10 most predominant ARGs in broiler samples (*n* = 24) included *sul*2 (95.8%, *n* = 23), *bla*TEM (91.7%, *n* = 22), *intI*1 (83.3%, *n* = 20), *sul*1 and *tet*M (79.2, *n* = 19), *tet*A (75%, *n* = 18), *aac*3-1a (66.7%, *n* = 16), *par*C (62.5%, *n* = 15), *sul*3 (50%, *n* = 12), and *bla*CTX-M (41%, *n* = 10), respectively. While in layer samples (*n* = 20), *bla*TEM (95%, n = 19), *intI1* (75%, *n* = 15), *sul2* (65%, *n* = 13), *aac*3-1a (60%, *n* = 12), *par*C (55%, *n* = 11), *tet*M (40%, *n* = 8), *tet*A and *sul*3 (30%, *n* = 6), *sul*1 (25%, *n* = 5), and *bla*CTX-M (20%, *n* = 4) were most frequent, respectively.

### Clustral analysis of shared resistance patterns among samples

**Fig. 5:**
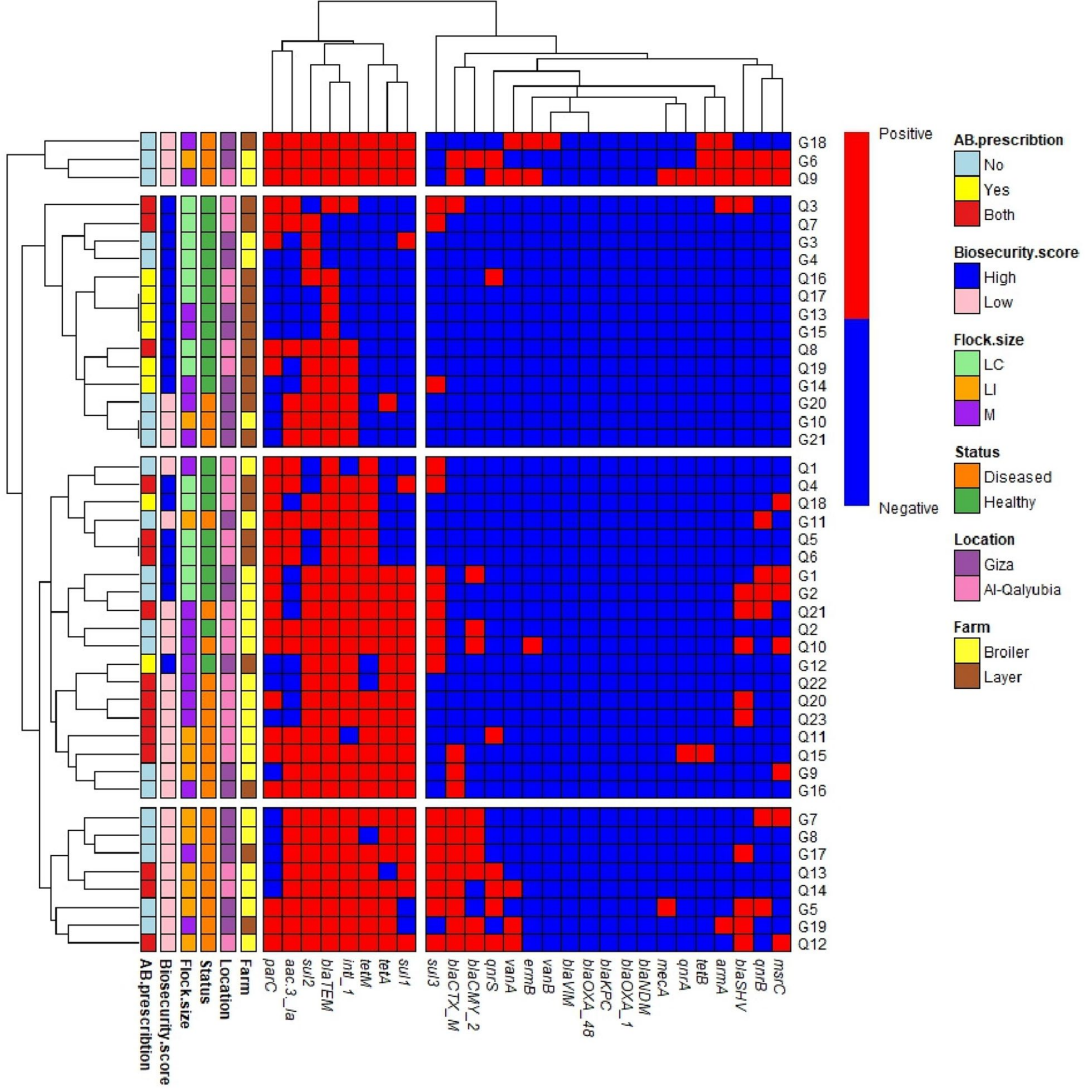
Hierarchically clustered heatmap of poultry screened samples from broilers and layers based on their antimicrobial resistance gene profiles. Blue indicates a negative result, while red indicates a positive result. Isolate clusters (rows) are labeled 1 to 4, and resistance gene groups (columns) are grouped into group 1 and group 2. • Flock size abbreviations: LC: Large commercial flocks5000-25,000 birds; LI: Large intensive scale > 25,000; M: medium scale 500-5000birds

The generated heatmap grouped the 44 pooled samples into four clusters (C1–C4) based on similar resistance gene patterns (Fig. 5). Two main gene groups were identified: Group G1, comprising highly prevalent genes (*parC*, *aac (3)−1a*, *sul*2, *bla*TEM, *intI*1, *tet*M, *tet*A, and *sul*1), and Group G2, containing lower-prevalence genes (*sul*3, *bla*CTX-M, *bla*CMY-2, *bla*SHV, *mec*A, *bla*OXA-1, *bla*OXA-48, *bla*KPC, *bla*VIM, *bla*NDM, *qnr*S, *qnr*A, *qnr*B, *van*A, *van*B, *tet*B, *arm*A, *erm*B, and *msr*C) (Fig. 5). Cluster 1 included only 3 pooled samples; all were diseased birds raised on low biosecurity farms with no antibiotic prescriptions. These pooled samples recorded positive PCR for all Group G1 genes, plus *tet*B, and *arm*A, indicating resistance to tetracyclines and aminoglycosides. Most Group G2 genes were frequently detected within this cluster. Two-thirds of these pooled samples were from broilers. Cluster 2, a large group (14 pooled samples), had the lowest resistance levels. None tested positive for carbapenemase genes (*bla*OXA-48, *bla*KPC, *bla*VIM, and *bla*NDM), vancomycin-resistant genes (*van*A, and *van*B*)*, macrolide-resistant genes (*erm*B, and *msr*C*)*, or other resistance genes like *bla*OXA-1, *bla*CMY-2, *tet*M, *tet*B, *qnr*A, and *qn*rB. The most common genes were *bla*TEM, *sul*2, and *intI*1, detected in 79% (*n* = 11), 71% (*n* = 10), and 50% (*n* = 7) of pooled samples, respectively. Most samples (79%, *n* = 11) came from healthy birds raised on high-biosecurity farms, primarily layers (81%, *n* = 9/11). Cluster 3 showed higher resistance. All samples in this cluster carried at least four Group G1 genes (50%). Additionally, 4 of the 19 samples (21.1%) were positive for all G1 genes, and 6 of the 19 samples (31.6%) were positive for 7 out of the 8 genes (87.5%). *bla*TEM was detected in all pooled samples. Carbapenem and vancomycin resistance genes were absent. Samples were from both healthy (9) and diseased (10) birds, mostly broilers (68%,* n* = 13) from low-biosecurity farms (92%, *n* = 12/13). Cluster 4 contained only diseased bird samples (100%, *n* = 8) from low-biosecurity, mainly large-scale broiler farms (75%,* n* = 6). All samples in this cluster (4) were positive for 6 or more genes (≥ 75%) out of the 8 genes belonging to Group G1 genes. At least 75% of samples (*n* = 6) were also positive for *tet*M, *tet*A, *sul*1, *sul*3, and *bla*CMY-2. Genes like *van*B, *erm*B, *qnr*A, and *tet*B were absent across all pooled samples within cluster 4.

### Class 1 integrons detection and co-occurrence with other ARGs

Class 1 integrons, specifically the *intI*1 gene encoding the integrase enzyme, promote lateral ARG transfer and integration of resistance genes [43, 44]. In this study, i*ntI*1 was detected in 35 out of 44 pools (79%). Notably, β-lactam resistance genes were found to co-occur in 100% (*n* = 35) of *intI*1-positive samples. High co-occurrence was also noted for sulphonamide (94.3%, *n* = 33), tetracyclines (77.1%, *n* = 27), quinolones and aminoglycoside (71.4%, *n* = 25). Glycopeptides (14.3%, *n* = 5) and macrolides (28.6, *n* = 10) had lower co-occurrence rates (Supplementary Figure SFig.3). Fisher’s exact test did not identify statistically significant associations between *intI1* and resistance to any antibiotic class at the conventional threshold (*p >* 0.05). However, descriptive examination of the data showed more frequent co-occurrence of *intI1* with resistance to β-lactams, sulphonamides, tetracyclines, aminoglycosides, and quinolones than with macrolide and glycopeptide resistance genes.

### Risk factors associated with the prevalence of ARGs (ARG richness metrics)

As shown in Table 4, a significantly higher mean ARG richness per sample was observed in diseased birds (mean = 10 ± 3.793, range = 4–15) compared to healthy birds (mean = 5.1 ± 3.177, range = 1–10) (*P* < 0.05, t-test). These diseased birds were raised on farms with low biosecurity scores (range = 2.5—4) and either did not follow antibiotic prescription guidelines or applied antibiotics inconsistently. ARG richness represents a valid metric for assessing the diversity of antimicrobial resistance genes (ARGs) within a sample. It is calculated as the total number of distinct ARGs detected per sample or group of samples [45]. A higher ARG richness indicates greater resistance diversity, which may indicate increased AMR risk and/or higher antibiotic exposure. The mean ARG richness of the samples taken from the governorates of Kalyoubia and Giza did not differ significantly. However, regarding poultry breed, Ross broilers exhibited the highest ARG richness (mean = 12 ± 5.85), followed by Cobb (mean = 10 ± 3.37), while among layers, Lohman white layer had higher ARG richness (mean = 9 ± 3.74) compared to other layer breeds. Moreover, samples from low-biosecurity farms showed a two-fold increase in ARG richness compared to high-biosecurity farms. Summer samples also had higher richness (mean = 9 ± 3.783) than winter samples (mean = 6 ± 4.48). Large intensive farms (> 25,000 birds; 10.4 ± 3.37) showed higher mean ARG richness compared to large commercial farms (5,000–25,000 birds; 5.3 ± 3.18). Higher mean ARG richness was also observed in farms using more than three antibiotic classes (9.85 ± 4.81) than in those using two classes (5.06 ± 2.57). Additionally, higher ARG richness was reported in farms reporting non-prescription (9.38 ± 4.53) or both prescription and non-prescription use (8.0 ± 3.07) compared to prescription-only use (3.25 ± 2.12). These patterns are presented descriptively due to non-independence of observations arising from multiple pools sampled within the same farm. Variables showing apparent differences in exploratory univariate analyses (production type, bird health, season, and biosecurity score) were subsequently included in a multivariable logistic regression to assess whether these trends persisted after adjustment. In the multivariable logistic regression analysis, where ARG richness was categorized as high (≥ 8 ARGs) or low (< 8 ARGs), none of the examined variables showed statistically significant associations (95% CI; *P >* 0.05; Supplementary Table 6).

## Discussion

AMR in poultry farming has become a serious threat to humans, animals and the entire ecosystem, highlighting the urgent need for interventions and solutions. Culture-independent methods, like PCR-based screening, are rapid and comprehensive methods used to detect ARGs from farms directly, regardless of the bacterial species present [21]. In Egypt, AMR in poultry and its products has been thoroughly investigated; however, the majority of studies have focused on particular bacterial pathogens such as *E. coli*, *Klebsiella* spp., and *Enterococcus* spp., using phenotypic culture-based methods [46]. To date and to the best of our knowledge, no records from Egypt or the Middle East have documented the use of direct PCR-based screening to assess ARG patterns in poultry farms, despite its affordability and application in other countries such as Italy [21, 47]. Notably, there are a few local studies that bypassed the isolation step and used direct PCR to detect specific hard-to-culture pathogens present in Egyptian poultry farms, as *Campylobacter* and *Mycoplasma* and their associated ARGs, but overlooked the rest of the potential community [22, 48]. Thus, our study is the first in Egypt to apply a low-cost, species-independent PCR approach to screen for resistance to seven antimicrobial classes and one mobile gene class in poultry farms from 9 farms in two governorates. Additionally, our study evaluated potential farm level risk factors and management practices that act as key drivers influencing resistance dynamics and ARG dissemination within these 9 poultry farms.

Based on our questionnaire analysis, the majority of the surveyed farms in Kalyoubia and Giza were deep litter-based systems (77%, *n* = 7/9), less than one month of age (47.7%, *n* = 21/44) and operating as medium-scale farms (44.4%, *n* = 18/44) (rearing 500 to 5,000 birds), which aligns with typical production systems in these regions [42, 49, 50]. Likewise, a study conducted [42] on six major enterprises in Kalyoubia reported that most poultry farms were ground-based and reared around 5,000 birds. The deep litter system is especially common in Kalyoubia, primarily due to financial constraints as 90% of farms in the region rely on loans and rented facilities, making deep litter a more affordable and practical option [42].

Our survey showed that there were more broiler farms in Kalyoubia (60%, *n* = 3/5), which supports what has already been said about the region's dominance in broiler production [49]. As for layer farms, we included a similar number from both Kalyoubia (50%, *n* = 2/4) and Giza (50%, *n* = 2/4), although earlier reports noted a higher concentration in layer farms in Giza. This difference likely reflects our sampling strategy aiming for balanced comparison rather than a deviation from regional production patterns. Furthermore, every broiler and layer farm reported taking antibiotics during its rearing cycle. This is consistent with earlier findings in Egypt [6, 9] and other countries, including Malaysia and Cameroon, where 80–100% of poultry farms were found to use antibiotics at different stages of production [51, 52].

Similarly to [53], we found that the overall usage of antimicrobials was higher in broiler farms compared to layer farms, likely due to the common use of antibiotics in broilers to enhance growth and feed efficiency [54]. However, lower usage in layers may be explained by two key factors. First, most layer farms in our study followed strong biosecurity measures (75%,* n* = 3/4) and relied on veterinary prescriptions (62.5%, *n* = 2/3). Previous studies have stated that improved biosecurity reduces the need for antimicrobial use, thereby lowering the risk of disease and antimicrobial-resistant bacteria [13, 55]. This aligns with our findings, which showed a positive correlation between higher biosecurity scores and the use of vet-guided prescriptions. Second, some farmers may limit antibiotic use in layers to avoid drug residues in eggs [56]. Of note, we also observed that broiler farms reported high usage of β-lactams, tetracyclines, and macrolides, consistent with reports from Egypt, Pakistan, and Germany, where these antibiotics are commonly used to treat enteric and respiratory diseases and support rapid growth [57, 58]. As for layer farms, they reported β-lactams, macrolides, and quinolones as the most frequent. In Bangladesh, fluoroquinolones (ciprofloxacin and enrofloxacin) and β-lactams (amoxicillin) were the most commonly utilized antimicrobials in 120 layer farms [59]. Similarly, in Greece, all *E. coli* isolates from layers displayed resistance to β-lactams (oxacillin) and macrolides, indicating common field use [60]. This is especially worrisome, because the WHO considers fluoroquinolones and macrolides to be extremely important antimicrobials for human health. Their misuse in poultry poses a serious risk of resistance transfer to humans, especially through foodborne pathogens [61].

Another striking observation was that two-thirds of the farms used antibiotics irregularly or without veterinary consultation, while one-third of the farms used three or more antibiotics in a single production cycle. Similar patterns of antibiotic misuse in poultry farms have been reported in countries like Kenya, and Grenada [62, 63]. Possible reasons for such misuse could be attributed to lack of awareness, availability of over-the-counter antibiotics, and high costs of veterinary services [6]. These findings highlight the urgent need for antimicrobial stewardship (AMS) programs and stronger surveillance of antimicrobial use and AMR in poultry production.

Regarding targeted resistome PCR-based screening, the most predominant ARGs were those that encoded resistance to β-lactam, sulphonamides, and tetracyclines, with *bla*TEM, *sul*2, *intI*1, *aac*3, and *tet*M identified as the five most frequently recorded genes, respectively, across all the screened pooled samples. Therefore, these genes are widely detected in poultry farms, highlighting poultry as a significant reservoir for clinically important bacterial resistance genes [64]. Reflecting similar findings, a Nigerian study also reported the dominance of the blaTEM gene in fecal poultry isolates, which is known to confer resistance to penicillin and first-generation cephalosporins [65]. Likewise, direct PCR screening of poultry litter samples in Northern Brazil revealed a high prevalence of β-lactams, sulphonamides and tetracyclines resistance genes, with *tet*M, *gyr*A, *bla*TEM, *erm*B, and *sul*−1 as the most prevalent in the analyzed samples [66]. In accordance with several studies reporting low to undetectable levels of carbapenemase genes (*bla*OXA-48, *bla*NDM, *bla*VIM, *bla*KPC) and *bla*OXA-1, our study reported a complete absence of these genes [21, 47]. Their absence likely relates to carbapenems being last-resort antibiotics used only in humans and banned in animals in most countries, including Europe [67].

Interestingly, sulphonamide-resistance genes (*sul*1, *sul*2, and *sul*3) were highly prevalent despite the fact that only 22% of farms used sulphonamide drugs for treatment or prophylaxis. A possible reason is that sulphonamides are old antibiotics widely used in poultry farms for decades, with long half-lives that promote their persistence in the environment [68, 69]. Other possible reasons include poor documentation of antimicrobial use and the rapid spread of *sul*1 and *sul*2 through horizontal gene transfer, as these genes are commonly located on mobile genetic elements such as class 1 integrons and plasmids, respectively [11, 69, 70]. Among the three sulphonamide-resistance genes, *sul*2 was the most dominant, in agreement with [64] and other studies [71]. As for tetracycline-resistance genes, *tet*M showed the highest prevalence in our study compared to other tetracycline-resistance genes, likely due to its widespread presence among natural bacterial species in the chicken fecal microbiota [21].

Furthermore, high ARG richness in poultry farms was observed with several risk factors, including the use of Ross (mean ARGs:11.75 ± 5.85) and Cobb broiler breeds (mean ARGs: 10.45 ± 3.37), Lohmann White layers (mean ARGs: 9 ± 3.74), diseased birds (mean ARGs: 10 ± 3.793), low biosecurity scores (mean ARGs: 9.85 ± 3.77), intensive large-scale flocks (mean ARGs: 10.4 ± 3.37), lack of prescriptions (mean ARGs: 9.38 ± 4.53), and the use of more than three antibiotics (mean ARGs: 9.85 ± 4.81) as shown in Table 4. Notably, although high ARGs richness was noticed between these variables, multivariate logistic regression analysis showed no significant difference (CI = 95%; *P >* 0.05), (Supplementary Table 6). Several studies have also shed light on how variable farm characteristics and biosecurity measures contribute to the dissemination of ARGs in poultry farms [52, 72].

In our study, broilers exhibited higher levels of resistance genes than layers, consistent with our antimicrobial use findings, as broilers received more antibiotics based on the questionnaire data. This observation aligns with existing evidence that increased antimicrobial use imposes selection pressure, thereby promoting higher resistance gene prevalence [73].

Heatmap-based cluster analysis showed that *par*C, *sul*1, *sul*2, *tet*M, *intI*1, and *aac* (3)−1a were the most prevalent ARGs, mainly linked to broiler farms and low biosecurity. This supports the role of poor biosecurity in promoting ARG spread [13]. Their pattern and prevalence in unhygienic settings also suggest association with mobile genetic elements like plasmids and integrons. For instance, while both *sul*1 and *sul*2 confer resistance to sulphonamides, they differ in genetic context: *sul*1 is typically associated with class 1 integrons, whereas *sul*2 is more often plasmid-mediated [74]. Their co-occurrence with *intI*1 suggests active gene transfer and integron mobilization. Similarly, *tet*M is frequently found on conjugative transposons like Tn5397-like and Tn916-like, which could account for its high frequency in broiler environments exposed to antibiotics [75]. Moreover, the presence of plasmid-mediated fluoroquinolone resistance genes (e.g., *qnr*) and *par*C in broilers suggests that resistant strains are actively circulating, particularly in intensive systems with inadequate biosecurity [76]. Several resistance genes were frequently co-associated with the *intI*1 gene, such as β-lactams, sulphonamides, tetracyclines, aminoglycosides, and quinolones, indicating that the *intI1* gene was highly prevalent in poultry farms. According to [64], this suggests that the poultry resistome are associated with mobile genetic elements, indicating a high risk of horizontal transmission to humans through commensal bacteria or foodborne pathogens, posing a significant risk to public health. However, our study did not find any significant association between *intI*1 and any resistance genes using Fisher's Exact Test, (*p >* 0.05). The absence of statistically significant associations may be due to limited sample size and low cell counts within contingency tables, which reduce statistical power. Nevertheless, the co-occurrence of *intI*1 and glycopeptide resistance (14.3%, *n* = 5) or macrolide (28.6%, *n* = 10) resistance genes were least observed. This suggests that resistance to these classes may be primarily caused by chromosomal mutations or plasmid-mediated genes independent of integrons, such as *erm* for macrolides and *van* genes for glycopeptides [77]. These findings are consistent with previous reports demonstrating that *intI*1 is more strongly associated with resistance to sulphonamides, aminoglycosides, and β-lactams rather than glycopeptides [74]. Also, direct systematic links between *intI*1 and *van* or erm genes are comparatively sparse [78].

*Overall, although our study* provides preliminary insights for AMR in 9 poultry farms in Kalyoubia and Giza, several limitations are still present. First, the study included a limited number of farms from only two governorates, which may not fully capture the geographic diversity of poultry production in Egypt. Second, the use of pooled samples means our findings reflect the farm-level presence and co-occurrence of resistance genes, not their co-localization within individual bacterial genomes. Although this distinction should be noted, as it could technically overestimate co-occurrence compared to isolate-level analysis, our primary objective was to obtain a rapid, feasible, and high-throughput snapshot of the most prevalent ARGs circulating in poultry farms. This methodology, employing uniform pooling of samples from birds with analogous status and exposure conditions, was intentionally selected to achieve a robust assessment of the overall antimicrobial resistance reservoir in the study environment. Another limitation is that the newly designed sulfonamide resistance primers were not validated by amplicon sequencing or an extended cross-reactivity panel. However, in-silico screening, clean amplification profiles (Supplementary file 2), and consistently high detection of *sul2* suggests that the observed pattern is biologically consistent with other studies [64, 71, 79] and support their specificity. Finally, although the fecal composition and microbiome of broilers and layers differ, and could theoretically influence PCR efficiency, the DNA extraction quality was consistently high across all pooled samples (Supplementary Table 1). Uniform laboratory procedures and a standardized extraction protocol were applied to all samples, and both positive and negative controls were included in each run. These measures suggest that any bias in ARG detection due to pooling or production type is minimal.

## Conclusion

This study is the first in Egypt and the Middle East to use direct PCR for detecting antibiotic resistance genes (ARGs) in poultry, providing a rapid, low-cost, and non-invasive method for AMR surveillance. Our findings offer early insights into the poultry resistome and highlight differences between broilers and layers under medium and intensive production in 9 poultry farms. While limited in geographic scope, the study demonstrates the utility of molecular tools for monitoring resistance and paves a way for other researchers to use a simple practical method for evaluating AMR in poultry farms in high poultry producing areas as Kalyoubia and Giza in Egypt and other LMICs. Of note, due to Egypt’s regional diversity and limited documentation records, an urgent more representative AMR surveillance across additional Egyptian governorates is required. Additionally, these results emphasize the need for broader, integrated surveillance, including other farming systems, and support a One Health approach to tackling AMR at the animal-human–environment interface.

## Supplementary Information

Supplementary Material 1.

Supplementary Material 2.

Supplementary Material 3.

## Data Availability

All data supporting the findings of this study are available within the article.
