# Supplementary material for: Antimicrobial resistance profiling in poultry industry: a culture-independent resistome analysis and risk factor assessment
Source: BMC Vet Res. 2026 Mar 14;22:212. doi: 10.1186/s12917-026-05334-w (PMC13063522; doi:10.1186/s12917-026-05334-w)
Supplement: Supplementary file 1 — Supplementary Material 1. [file 12917_2026_5334_MOESM1_ESM.zip › MH_Supplementary_Tables_20251206.pdf]

Antimicrobial Resistance Profiling In Poultry Industry: A Culture-Independent Resistome Analysis and Risk Factor Assessment

Sabah Ali[a*], Mariam Hassan[b,c*], Tamer Essam[b], Shimaa Abdel malik[a], Khaled F. Al-Amry[a*]

[a]*Department of Microbiology, Faculty of Veterinary Medicine, Cairo University, Giza, Egypt.*

[b]*Department of Microbiology and Immunology, Faculty of Pharmacy, Cairo University, Cairo, Egypt.*

[c]*Department of Microbiology and Immunology, Faculty of Pharmacy, Galala University, New Galala City, Suez, Egypt*

Supplementary Tables

Supplementary Table 1 (ST1). DNA concentration and purity measurements of all 44 pooled fecal samples, assessed using Nanophotometer.

Sample code	Q1	Q2	Q3	Q4	Q5	Q6	Q7	Q8	Q9	Q10	Q11
DNA Conc (ng/µL) ± SD*	215.6 ±30.8	489.2 ± 46	720.5 ± 10	304.7 ±25.1	856.3 ±17.3	635.1 ±14.6	425.4 ±19.3	509.9 ±27.2	311.2 ±29.8	690.7 ±36.9	222.4 ±30.96
Purity (A260/A280) ± SD	1.78 ±0.08	1.92 ±0.08	2.01 ±0.02	1.66 ±0.01	2.08 ±0.02	1.9 ±0.08	1.71 ±0.02	2 ±0.04	1.55 ±0.09	2.03 ±0.05	167 ± 30.07
Sample code	Q12	Q13	Q14	Q15	Q16	Q17	Q18	Q19	Q20	Q21	Q22
DNA Conc (ng/µL) ± SD	222.4 ±30.9	777.6 ±44	350.3 ±20	598.8 ±53.9	721.4 ±11.3	278.9 ±43.5	463 ±30.8	842.2 ±37.9	312.5 ±17.0	505.6 ±19.9	794.1 ±50.0
Purity (A260/A280) ± SD	1.67 ±0.07	2.1 ±0.04	1.8 ±0.06	1.94 ±0.08	2.07 ±0.01	1.59 ±0.07	1.85 ±0.09	2 ±0.07	1.72 ±0.03	1.88 ±0.07	2.09 ±0.02
Sample code	Q23	G1	G2	G3	G4	G5	G6	G7	G8	G9	G10
DNA Conc (ng/µL) ± SD	401.7 ±58.4	335.6 ±14.5	538.3 ±19.3	473 ±27	467.7 ±31.9	766.4 ±11.8	654.6 ±30.6	774.4 ± 20.2	682.4 ± 11	930.1 ± 16.2	355.6 ± 13
Purity (A260/A280) ± SD	1.6 ±0.05	1.73 ±0.03	2.04 ±0.03	2.14 ±0.02	2.05 ±0.01	1.86 ±0.04	1.82 ± 0.03	1.65 ± 0.03	1.73 ± 0.01	1.74 ± 0.02	1.56 ± 0.03
Sample code	G11	G12	G13	G14	G15	G16	G17	G18	G19	G20	G21
DNA Conc (ng/µL) ± SD	577.2 ± 12.4	668.5 ± 8.8	524.5 ± 7.5	826 ± 11	935 ± 10.5	746.5 ± 10.8	661 ± 23.1	927.7 ± 12.8	451.1 ± 11.7	545 ± 7.8	347.1 ± 22.9
Purity (A260/A280) ± SD	1.85 ± 0.04	2.03 ± 0.02	1.92 ± 0.02	1.87 ± 0.02	2.06 ± 0.02	2.1 ± 0.02	2.04 ± 0.03	1.84 ± 0.03	1.76 ± 0.04	1.45 ± 0.04	2.08 ± 0.02

*SD: Standard deviation of means.

Supplementary Table 2 (ST2): Distribution of samples according to health status and farm category

Governorates	Farm category	Health status	No of samples	No of pools (%)	Farm codes
Kalyoubia	Broiler	Apparently healthy	10	2 (4.5)	FB1
		Diseased	55	11 (25)	FB1, FB2, FB3
	Layer	Apparently healthy	50	10 (22.7)	FL1, FL2
		Diseased	0	0	0
Giza	Broiler	Apparently healthy	20	4 (9.1)	FB4
		Diseased	35	7 (15.9)	FB5
	Layer	Apparently healthy	20	4 (9.1)	FL3
		Diseased	30	6 (13.6)	FL4
**Total**			220	44	9 farms

Supplementary Table 3 (ST3): Distribution of Medications by Class and Generic Name Across Broiler and Layer Farms

**Antibiotic class**	**Examples (Generic name) and dose for both Broilers and Layers**	**Farm code and count**	**Frequency & Percentage (%) per total farms**	**Frequency & Percentage in (%) Broiler vs layer farms**
B lactam	Amoxicillin (10-20mg/kg/3-5 days) (DW)	FL1, FL2, FL3, FB4, FB5 (n=5)	7/9 (77.9)	4/5 (80) vs 3/4 (75)
	Cephalosporin (cephotax) (5-10mg/kg/3-5 days) (INJ)	FB1, FB2 (n=2)		
Tetracyclines	Oxytetracycline (40-60mg/kg/3-5days) (DW)	FB1 (n=1)	4/9 (44.4)	4/5 (80) vs 0/4 (0)
	Tetracycline (40-60mg/kg/3-5days) (DW)	FB2, FB3, FB4 (n=3)		
Fluroquinolones	Enrofloxacin (5-10mg/kg/3-5 days) (DW)	FL1, FB2, FB4 (n=3)	3/9 (33.3)	2/5 (40) vs ¼ (25)
Macrolides	Spiramycin (38-50mg/kg/3-5 days) (DW)	FB1 (n=1)	6/9 (66.6)	4/5 (80) vs 2/4 50
	Tylosine (50mg/kg/3-5 days)/ Telmicosil (12-25mg/kg/3-5 days) (DW)	FB1, FB3, FB5, FL2, FL3 (n=5)		
Aminoglycoside	Kanamycin (15mg/kg/3-5days) (INJ)	FB1 (n=1)	2/9 (22.2)	2/5 (40) vs 0/4 (0)
	Streptomycin (50mg/kg/3-5days) (DW)	FB2 (n=1)		
Polymyxin	Colistin 2.5-3mg/kg/3-5 days) (DW)	FB1 (n=1)	1/9 (11.1)	1/5 (20) vs 0/4 (20)
Lincosamide	Lenspectin (10mg/kg/3-5 days) (DW)	FB2 (n=1)	1/9 (11.1)	1/5 (20) vs 0/4 (0)
Florfenicol	Chloramphenicol (25-50mg/kg/3-5 days)	FL4 (n=1)	2/9 (22.2)	1/5 (20) vs ¼ (25)
	Deflobiotic (25-50mg/kg/3-5 days)	FB3 (n=1)		
Sulphonamides	Sulphamix (60-110mg/kg/5days) (DW)	FB5, FL4 (n=2)	2/9 (22.2)	1/5 (20) vs ¼ (25)
Vaccines	(pox, influenza, NDV, lasota, gumbro)	FB1, FB2, FB3, FB4, FB5, FL1, FL2, FL3, FL4 (n=9)	9/9 (100)	5/5 (100) vs 4/4 (100)
Antitoxins	IMA	FB1, FB5 (n=2)	2/9 (22.2)	2/5 (40) vs 0/4 (0)
Immune stimulant	-	FB1 (n=1)	1/9 (11.1)	1/5 (20) vs 0/4 (0)

- Total number of farms= 9, total broiler farms= 5, total layer farms= 4
- DW: Drinking Water, INJ: Injection.

Supplementary Table 4 (ST4): Detection of 27 Antimicrobial Resistance Genes (ARG) in Giza Samples by Qualitative PCR.

- Giza pools= 21

		Beta lactam					Tetracycline			Sulphonamides			Quinolones				Glycopeptides		Aminoglycosides		Integrase	Macrolides	
		bla CTXM	bla TEM	bla SHV	CMY2	mec A	tet A	tet B	tet M	sul 1	sul3	sul 2	qnr A	qnr B	qnr S	parc A	van A	van B	armA	aac3-la3	intl-1	ermB	msrC
G1	FB4	-	+	-	+	-	+	-	+	+	+	+	-	+	-	+	-	-	-	-	+	-	+
G2	FB4	-	+	+	-	-	+	-	+	+	+	+	-	+	-	+	-	-	-	-	+	-	+
G3	FB4	-	-	-	-	-	-	-	-	+	-	+	-	-	-	+	-	-	-	-	-	-	-
G4	FB4	-	-	-	-	-	-	-	-	-	-	+	-	-	-	-	-	-	-	-	-	-	-
G5	FB5	+	+	+	-	+	+	-	+	-	+	+	-	+	+	+	-	-	-	+	+	-	-
G6	FB5	+	+	+	+	-	+	+	+	+	-	+	-	+	+	+	-	-	+	+	+	-	+
G7	FB5	+	+	-	+	-	+	-	+	+	+	+	-	+	-	-	-	-	-	+	+	-	+
G8	FB5	+	+	-	+	-	+	-	-	+	+	+	-	-	-	-	-	-	-	+	+	-	-
G9	FB5	+	+	-	-	-	+	-	+	+	-	+	-	-	-	-	-	-	-	+	+	-	+
G10	FB5	-	+	-	-	-	-	-	-	-	-	+	-	-	-	-	-	-	-	+	+	-	-
G11	FB5	-	+	-	-	-	-	-	+	-	-	+	-	+	-	+	-	-	-	+	+	-	-
G12	FL3	-	+	-	-	-	+	-	-	+	+	+	-	-	-	-	-	-	-	-	+	-	-
G13	FL3	-	+	-	-	-	-	-	-	-	-	-	-	-	-	-	-	-	-	-	-	-	-
G14	FL3	-	+	-	-	-	-	-	-	-	+	+	-	-	-	-	-	-	-	-	+	-	-
G15	FL3	-	+	-	-	-	-	-	-	-	-	-	-	-	-	-	-	-	-	-	-	-	-
G16	FL4	+	+	-	-	-	+	-	+	+	-	+	-	-	-	+	-	-	-	+	+	-	-
G17	FL4	+	+	+	+	-	+	-	+	+	+	+	-	-	-	-	-	-	-	+	+	-	-
G18	FL4	-	+	-	-	-	+	+	+	+	-	+	-	-	-	+	+	+	+	+	+	+	-
G19	FL4	+	+	+	+	-	+	-	+	-	-	+	-	-	-	+	+	-	+	+	+	-	-
G20	FL4	-	+	-	-	-	+	-	-	-	-	+	-	-	-	-	-	-	-	+	+	-	-
G21	FL4	-	+	-	-	-	-	-	-	-	-	+	-	-	-	-	-	-	-	+	+	-	-

**blaOXA-1,bla*OXA-48, *bla*KPC, *bla*VIM, and *bla*NDM were negative in all samples , thus weren't included in table and only 25 ARGs with diverse profiles within samples are shown

Supplementary Table 5 (ST5): Detection of 27 Antimicrobial Resistance Genes (ARG) in Giza Samples by Qualitative PCR.

- Kalyoubia pools= 23

		Beta lactam					Tetracycline			Sulphonamides			Quinolones				Glycopeptides		Aminoglycosides		Integrase	Macrolides	
		bla CTXM	bla TEM	bla SHV	CMY2	Mec A	tet A	tet B	tet M	sul 1	sul3	sul 2	qnr A	qnr B	qnr S	parc A	van A	van B	armA	Aac3-Ia	intI-1	ermB	mcrC
Q1	**FB1**	-	+	-	-	-	-	-	+	-	+	-	-	-	-	+	-	-	-	+	-	-	-
Q2	**FB1**	-	+	-	+	-	+	-	+	+	+	+	-	-	-	+	-	-	-	+	+	-	-
Q3	**FL1**	+	+	+	-	-	-	-	-	-	+	-	-	-	-	+	-	-	+	+	+	-	-
Q4	**FL1**	-	+	-	-	-	-	-	+	+	+	-	-	-	-	+	-	-	-	+	+	-	-
Q5	**FL1**	-	+	-	-	-	-	-	+	-	-	-	-	-	-	+	-	-	-	+	+	-	-
Q6	**FL1**	-	+	-	-	-	-	-	+	-	-	-	-	-	-	+	-	-	-	+	+	-	-
Q7	**FL1**	-	-	-	-	-	-	-	-	-	+	+	-	-	-	+	-	-	-	+	-	-	-
Q8	**FL1**	-	+	-	-	-	-	-	-	-	-	+	-	-	-	+	-	-	-	+	+	-	-
Q9	**FB1**	+	+	+	-	+	+	+	+	+	-	+	+	+	+	+	+	-	+	+	+	+	+
Q10	**FB1**	-	+	+	+	-	+	-	+	+	+	+	-	-	-	+	-	-	-	+	+	+	+
Q11	**FB2**	-	+	-	-	-	+	-	+	+	-	+	-	-	+	+	-	-	-	+	-	-	-
Q12	**FB2**	+	+	+	+	-	+	-	+	+	+	+	-	-	+	+	+	-	-	+	+		+
Q13	**FB2**	+	+	-	+	-	-	-	+	+	+	+	-	-	+	-	-	-	-	+	+	-	-
Q14	**FB2**	+	+	-	-	-	+	-	+	+	+	+	-	-	+	-	+	-	-	+	+	-	-
Q15	**FB2**	+	+	-	-	-	+	+	+	+	-	+	+	-	-	+	-	-	-	+	+	-	-
Q16	**FL2**	-	+	-	-	-	-	-	-	-	-	+	-	-	+	-	-	-	-	-	-	-	-
Q17	**FL2**	-	+	-	-	-	-	-	-	-	-	-	-	-	-	-	-	-	-	-	-	-	-
Q18	**FL2**	-	+	-	-	-	-	-	+	-	-	+	-	-	-	+	-	-	-	-	+	-	+
Q19	**FL2**	-	+	-	-	-	-	-	-	-	-	+	-	-	-	+	-	-	-	-	+	-	-
Q20	**FB3**	-	+	+	-	-	+	-	+	+	-	+	-	-	-	+	-	-	-	-	+	-	-
Q21	**FB3**	-	+	+	-	-	+	-	+	+	+	+	-	+	-	+	-	-	-	-	+	-	-
Q22	**FB3**	-	+	-	-	-	+	-	-	+	-	+	-	-	-	-	-	-	-	-	+	-	-
Q23	**FB3**	-	+	+	-	-	+	-	+	+	-	+	-	-	-	-	-	-	-	-	+	-	-

**bla*OXA-1, *bla*OXA-48, *bla*KPC, *bla*VIM, and *bla*NDM were negative in all samples , thus weren't included in table and only 25 ARGs with diverse profiles within samples are shown

Supplementary Table 6 (ST6): Multivariate logistic regression analysis of factors associated with ARG richness in poultry farms.

Variable	Category	OR	95% CI	*p*-value
**Production**	Layer	1	Reference	–
	Broiler	2.58	0.47–14.33	0.265
**Status**	Apparently healthy	1	Reference	–
	Dead (Diseased)	1.26	0.03–62.17	0.901
**Season**	Winter	1	Reference	–
	Summer	2.21	0.21–22.06	0.475
**Biosecurity score**	High	1	Reference	–
	Low	5.74	0.14–264.39	0.330

Total farms = 9, total pools = 44
OR = odds ratio; 95% CI = 95% confidence interval.
Reference categories are indicated with OR = 1.
Significance was set at $p < 0.05$.
ARG richness was dichotomized based on the median value of 8: “High” (1) for samples with ARG counts above 8, and “Low” (0) for samples with ARG counts ≤ 8. Out of 44 samples, 25 were classified as Low ARG and 19 as High ARG.
