# Supplementary material for: Antimicrobial resistance profiling in poultry industry: a culture-independent resistome analysis and risk factor assessment
Source: BMC Vet Res. 2026 Mar 14;22:212. doi: 10.1186/s12917-026-05334-w (PMC13063522; doi:10.1186/s12917-026-05334-w)
Supplement: Supplementary file 1 — Supplementary Material 1. [file 12917_2026_5334_MOESM1_ESM.zip › MH_SA_Supplementary_Figures_20250913.pdf]

Antimicrobial Resistance Profiling In Poultry Industry: A Culture-Independent Resistome Analysis and Risk Factor Assessment

Sabah Ali[a*], Mariam Hassan[b,c*], Tamer Essam[b], Shaymaa Abdelmalek Mohamed[a], Khaled F. Al-Amry[a]

[a]*Department of Microbiology, Faculty of Veterinary Medicine, Cairo University, Giza, Egypt.*

[b]*Department of Microbiology and Immunology, Faculty of Pharmacy, Cairo University, Cairo, Egypt.*

[c]*Department of Microbiology and Immunology, Faculty of Pharmacy, Galala University, New Galala City, Suez, Egypt*

Supplementary Figures

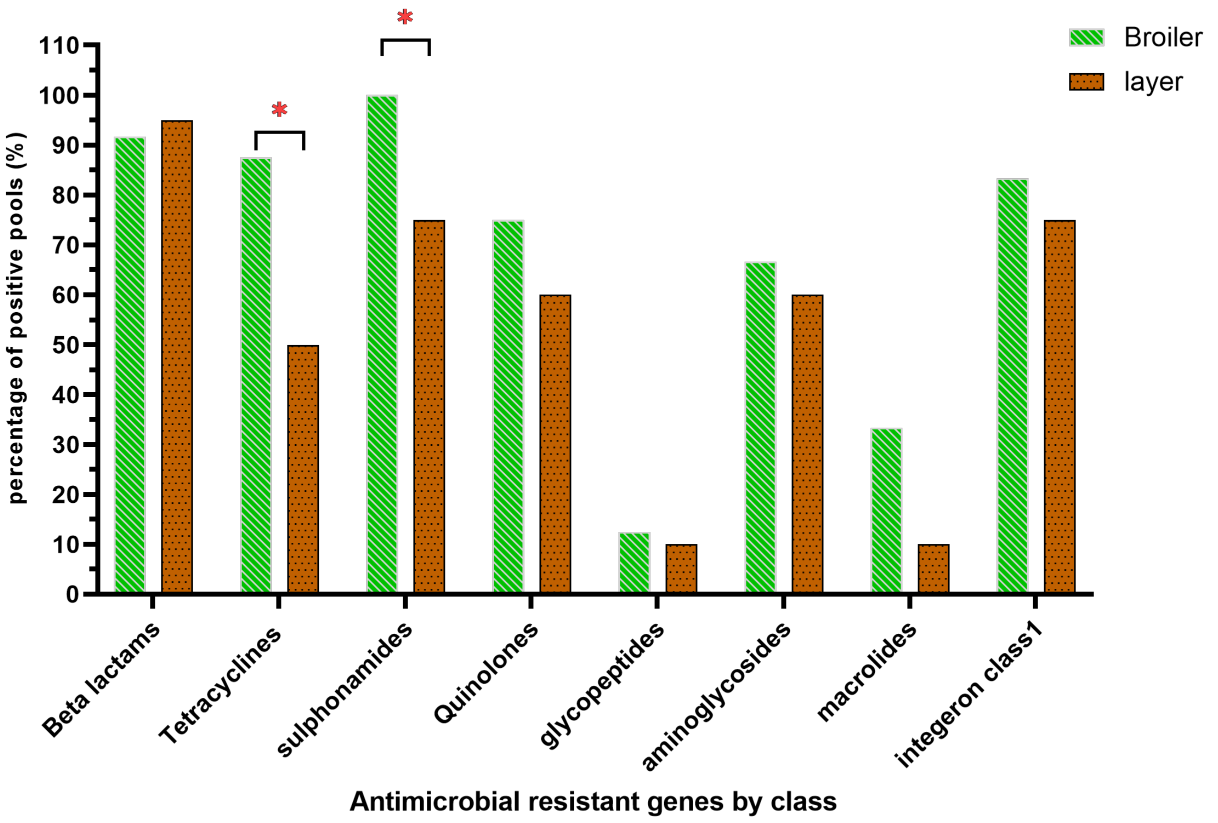

SFig.1: percent of postive pools for ARG by class across broiler and layer flocks

Supplementary Figure 1 (SFig.1): Percent of positive pools for ARG by class across broiler and layer flocks, total broiler pools n= 24 while total layer =20, asterisk (*) signifies significant differs in resistance per class between broiler and layers. Positive calculation was considered positive if any or all genes within class was detected in pool and marked (1); otherwise, if none was detected, it was marked (0)

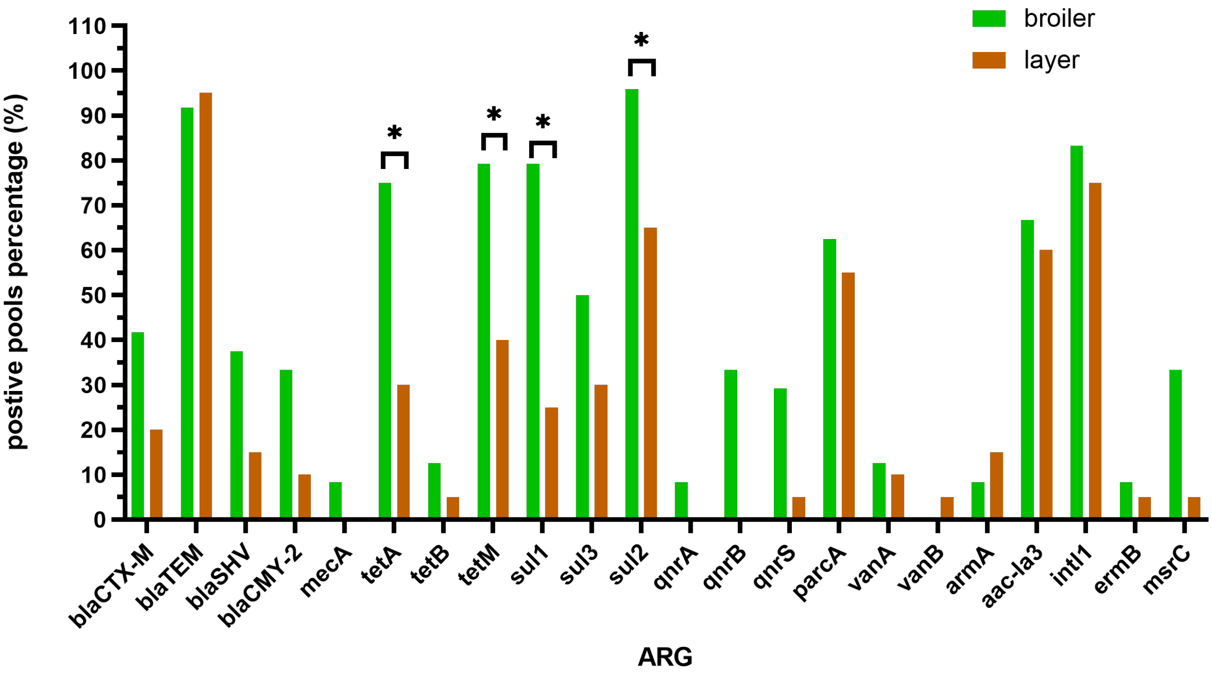

SFig.2: Relative abundance of ARGs among broiler and layer flocks

Supplementary Figure 2 (SFig.2): Relative abundance of ARGs among broiler and layer flocks. Asterisk (*) signifies significant differs in ARG between broiler and layers.

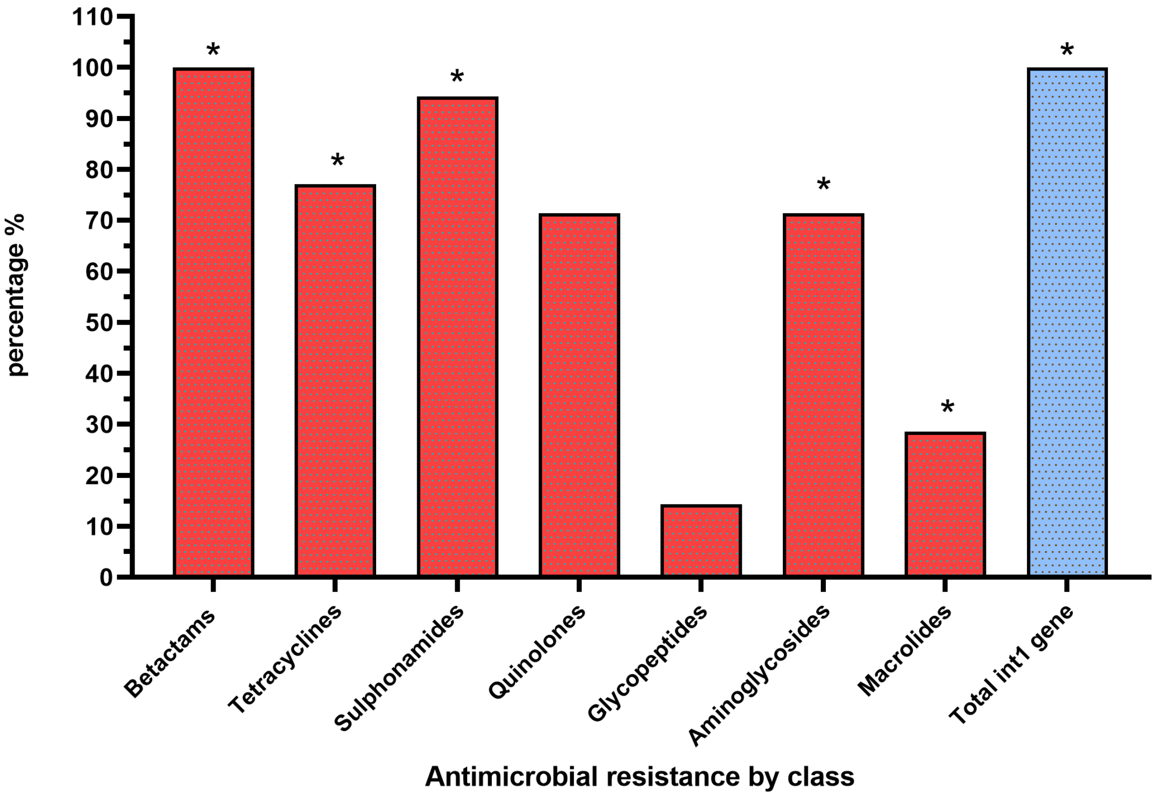

SFig.3: Relative co-occurance of *intl1* gene with other antimicrobial resistance classes

Supplementary Figure 3 (SFig.3): Relative co-occurrence of intl1 gene with other antimicrobial classes. N of intl1=, asterisk (*) indicates significant co-occurrence between *intl*1 gene and specific antimicrobial resistance class.
