# Supplementary material for: Antimicrobial resistance profiling in poultry industry: a culture-independent resistome analysis and risk factor assessment
Source: BMC Vet Res. 2026 Mar 14;22:212. doi: 10.1186/s12917-026-05334-w (PMC13063522; doi:10.1186/s12917-026-05334-w)
Supplement: Supplementary file 2 — Supplementary Material 2. [file 12917_2026_5334_MOESM2_ESM.pdf]

Antimicrobial Resistance Profiling In Poultry Industry: A Culture-Independent Resistome Analysis and Risk Factor Assessment

Sabah Ali[a*], Mariam Hassan[b,c*], Tamer Essam[b], Shimaa Abdel malik[a], Khaled F. Al-Amry[a*]

[a]*Department of Microbiology, Faculty of Veterinary Medicine, Cairo University, Giza, Egypt.*

[b]*Department of Microbiology and Immunology, Faculty of Pharmacy, Cairo University, Cairo, Egypt.*

[c]*Department of Microbiology and Immunology, Faculty of Pharmacy, Galala University, New Galala City, Suez, Egypt*

(Supplementary file 2)

Part 1 Insilco evaluation of our designed primers

Oligo evaluation of designed primers:

Gene name	primer	reference
***sul*1**	F: CGCACCGGAAACATCGCTGC<br>R: TGAAGTTCCGCCGCAAGGCT	Designed in our study
sul2	F: TCCGATGGAGGCCGGTATCTGG<br>R: CGGGAATGCCATCTGCCTTGAG	Designed in our study
sul3	F: AGTAGCTGCACCAATACGCT<br>R: CAACTGAAGTGGGCGTTGTG	Designed in our study

Gene name	primer	TM©	GC%	GC clamp	Length (bp)	2ry structure	Primer dimer	score
sul1	F: CGCACCGGAAACATCGCTGC	75	65	2	20	weak	none	Very good***
	R: TGAAGTTCCGCCGCAAGGCT	73	60	3	20	moderate	none	Good**
sul2	F: TCCGATGGAGGCCGGTATCTGG	74	63	2	22	moderate	none	Good **
	R: CGGGAATGCCATCTGCCTTGAG	73	59	1	22	none	none	Excellent ****
sul3	F: AGTAGCTGCACCAATACGCT	61	50	3	20	weak	none	Very good***
	R: CAACTGAAGTGGGCGTTGTG	66	55	1	20	moderate	none	Good **

Screenshots of evaluation of designed primers using OligoEvaluator (https://www.oligoevaluator.com/OligoCalcServle).

OVERALL: All primers showed excellent to good score, *sul*2 forward and reversed had best qualifications according to program.

***sul*1 forward oligo-evaluation:** very good primer with moderate length and weak ability to form 2ry structures and primer dimers, thus enhancing sensitivity.

1. Analysis Results

Sequence: 5' CGCACCGGAAACATCGCTGC 3'

Base Count	Molecular Weight	Extinction Coefficient	Oligo Type	μg/OD at 260 nm	Length (bp)	Tm (°C)	GC%	GC Clamp	Run Length (bp)	Secondary Structure	Primer Dimer	BLAST
A = 5, U = 0, G = 5, C = 8, T = 2, I = 0, Total = 20	6072.0	184.7	No Mod	32.9	20	75.1	65.0	2	3	Weak	No	Sequence

***sul*1 reverse oligo-evaluation:** good primer with moderate length and moderate ability to form 2ry structures and no ability to form primer dimers, thus enhancing sensitivity. Also, TM between both is less than 5C thus enhance amplicon generation.

1. Analysis Results

Sequence: 5' TGAAGTTCCGCCGCAAGGCT 3'

Base Count	Molecular Weight	Extinction Coefficient	Oligo Type	μg/OD at 260 nm	Length (bp)	Tm (°C)	GC%	GC Clamp	Run Length (bp)	Secondary Structure	Primer Dimer	BLAST
A = 4, U = 0, G = 6, C = 6, T = 4, I = 0, Total = 20	6118.1	185.6	No Mod	33.0	20	73.2	60.0	3	2	Moderate	No	Sequence

***sul*2 forward oligo-evaluation:** good primer with moderate length and ability to form 2ry structures and no primer dimers formation, thus enhancing sensitivity.

1. Analysis Results

Sequence: 5' TCCGATGGAGGCCGGTATCTGG 3'

Base Count	Molecular Weight	Extinction Coefficient	Oligo Type	μg/OD at 260 nm	Length (bp)	Tm (°C)	GC%	GC Clamp	Run Length (bp)	Secondary Structure	Primer Dimer	BLAST
A = 3, U = 0, G = 9, C = 5, T = 5, I = 0, Total = 22	6807.5	209.2	No Mod	32.5	22	74.8	63.6	2	2	Moderate	No	Sequence

***sul*2 reverse oligo-evaluation:** excellent primer with moderate length and no ability to form 2ry structures and primer dimers, thus enhancing sensitivity. Also, TM between both is less than 5C thus enhance amplicon generation.

1. Analysis Results

Sequence: 5' CGGGAATGCCATCTGCCTTGAG 3'

Base Count	Molecular Weight	Extinction Coefficient	Oligo Type	μg/OD at 260 nm	Length (bp)	Tm (°C)	GC%	GC Clamp	Run Length (bp)	Secondary Structure	Primer Dimer	BLAST
A = 4, U = 0, G = 7, C = 6, T = 5, I = 0, Total = 22	6751.5	204.5	No Mod	33.0	22	73.6	59.1	1	3	None	No	Sequence

***sul*3 forward oligo-evaluation:** very good primer with moderate length and weak ability to form 2ry structures and primer dimers, thus enhancing sensitivity.

1. Analysis Results

Sequence: 5' AGTAGCTGCACCAATACGCT 3'

Base Count	Molecular Weight	Extinction Coefficient	Oligo Type	µg/OD at 260 nm	Length (bp)	Tm (°C)	GC%	GC Clamp	Run Length (bp)	Secondary Structure	Primer Dimer	BLAST
A = 6, U = 0, G = 4, C = 6, T = 4, I = 0, Total = 20	6086.1	193.1	No Mod	31.5	20	61.5	50.0	3	2	Weak	No	Sequence

***sul*3 reverse oligo-evaluation:** good primer with moderate length and moderate ability to form 2ry structures and no ability to form primer dimers, thus enhancing sensitivity. Also, TM between both is less than 5C thus enhance amplicon generation.

1. Analysis Results

Sequence: 5' CAACTGAAGTGGGCGTTGTG 3'

Base Count	Molecular Weight	Extinction Coefficient	Oligo Type	µg/OD at 260 nm	Length (bp)	Tm (°C)	GC%	GC Clamp	Run Length (bp)	Secondary Structure	Primer Dimer	BLAST
A = 4, U = 0, G = 8, C = 3, T = 5, I = 0, Total = 20	6213.1	193.8	No Mod	32.1	20	66.7	55.0	1	3	Moderate	No	Sequence

Evaluation of designed primers specificity using primer blast and Insilco-PCR:

1A) *sul*1 primer set specificity evaluation using NCBI primer blast: (https://www.ncbi.nlm.nih.gov/tools/primer-blast/)

Overall: The newly designed *sul1* primer pair produced highly specific matches in the NCBI database at 162 bp, yielding 1,017 hits exclusively to *sul1* sequences across a wide range of bacterial species. These included *Pseudomonas* spp., *Klebsiella oxytoca*, *Escherichia coli*, *Enterobacter hormaechei* subsp. *steigerwaltii*, *Salmonella* spp., *Proteus* spp., among others, indicating its potential utility as a universal *sul1* primer for direct PCR across diverse bacterial taxa.

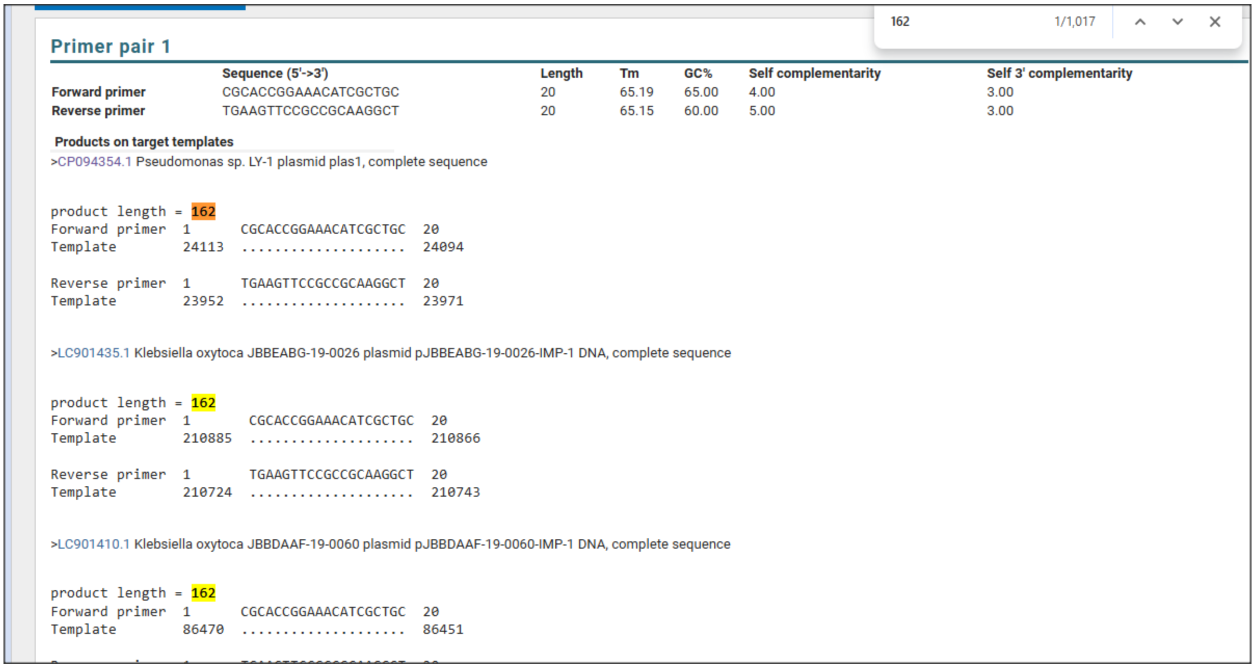

Primer pair 1

	Sequence (5'->3')	Length	Tm	GC%	Self complementarity	Self 3' complementarity
Forward primer	CGCACCGGAAACATCGCTGC	20	65.19	65.00	4.00	3.00
Reverse primer	TGAAGTTCCGCCGCAAGGCT	20	65.15	60.00	5.00	3.00

Products on target templates

>CP094354.1 Pseudomonas sp. LY-1 plasmid plas1, complete sequence

```
product length = 162
Forward primer  1      CGCACCGGAAACATCGCTGC  20
Template        24113  ....................  24094

Reverse primer  1      TGAAGTTCCGCCGCAAGGCT  20
Template        23952  ....................  23971
```

>LC901435.1 Klebsiella oxytoca JBBEABG-19-0026 plasmid pJBBEABG-19-0026-IMP-1 DNA, complete sequence

```
product length = 162
Forward primer  1       CGCACCGGAAACATCGCTGC  20
Template        210885  ....................  210866

Reverse primer  1       TGAAGTTCCGCCGCAAGGCT  20
Template        210724  ....................  210743
```

>LC901410.1 Klebsiella oxytoca JBBDAAF-19-0060 plasmid pJBBDAAF-19-0060-IMP-1 DNA, complete sequence

```
product length = 162
Forward primer  1      CGCACCGGAAACATCGCTGC  20
Template        86470  ....................  86451
```

1B) *sul*1 primer set specificity evaluation using Insilco-PCR amplification: http://insilico.ehu.es/PCR/Amplify.php)

Overall, in-silico PCR (insilico.ehu.es) demonstrated that the *sul1* primer pair produced the expected 162-bp amplicon in multiple reference bacterial genomes, including *Escherichia coli*, *Salmonella* spp., and *Klebsiella* spp., further supporting its specificity and suitability for broad detection of *sul1* across diverse taxa.

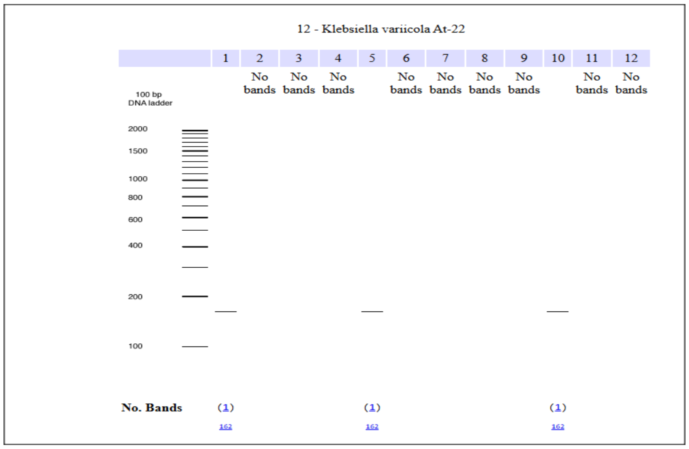

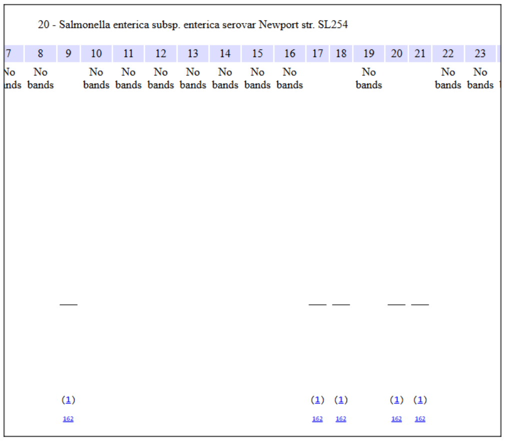

Klebsiella spp sul1 products

Salmonella sul1 products

2A) *sul*2 primer set specificity evaluation using NCBI primer blast: (https://www.ncbi.nlm.nih.gov/tools/primer-blast

Overall: The newly designed *sul2* primer pair produced highly specific matches in the NCBI database at 190 bp, yielding 955 hits exclusively to *sul2* sequences across a wide range of bacterial species. These *included Sphingopyxis spp, Glaesserella parasuis, Acinetobacter baumannii, Pasteurella multocida, Vibrio cholerae, Klebsiella spp*, *Escherichia coli*, *Salmonella* spp., *Proteus* spp., among others, indicating its potential utility as a universal *sul2* primer for direct PCR across diverse bacterial taxa.

	Sequence (5'->3')	Length	Tm	GC%	Self complementa[illegible]	
**Forward primer**	TCCGATGGAGGCCGGTATCTGG	22	65.61	63.64	4.00	
**Reverse primer**	CGGGAATGCCATCTGCCTTGAG	22	63.83	59.09	5.00	2.00

190 1/955

Products on target templates

>OZ368119.1 Sphingopyxis sp

```
product length = 190
Forward primer  1       TCCGATGGAGGCCGGTATCTGG  22
Template        475344  ......................  475365

Reverse primer  1       CGGGAATGCCATCTGCCTTGAG  22
Template        475533  ......................  475512
```

>CP041334.1 Glaesserella parasuis strain HPS412 chromosome, complete genome

```
product length = 190
Forward primer  1        TCCGATGGAGGCCGGTATCTGG  22
Template        1155264  ......................  1155285

Reverse primer  1        CGGGAATGCCATCTGCCTTGAG  22
Template        1155453  ......................  1155432


product length = 190
Forward primer  1        TCCGATGGAGGCCGGTATCTGG  22
Template        1142299  ......................  1142320

Reverse primer  1        CGGGAATGCCATCTGCCTTGAG  22
Template        1142488  ......................  1142467
```

>CP054237.1 Escherichia coli strain EcPF5 plasmid p1, complete sequence

2B) *sul*2 primer set specificity evaluation using Insilco-PCR amplification: http://insilico.ehu.es/PCR/Amplify.php)

Overall, in-silico PCR (insilico.ehu.es) demonstrated that the *sul2* primer pair produced the expected 190-bp amplicon in multiple reference bacterial genomes, including *Acinetobacter spp.*, *Escherichia coli*, *Salmonella* spp., and *Klebsiella* spp., further supporting its specificity and suitability for broad detection of *sul2* across diverse taxa.

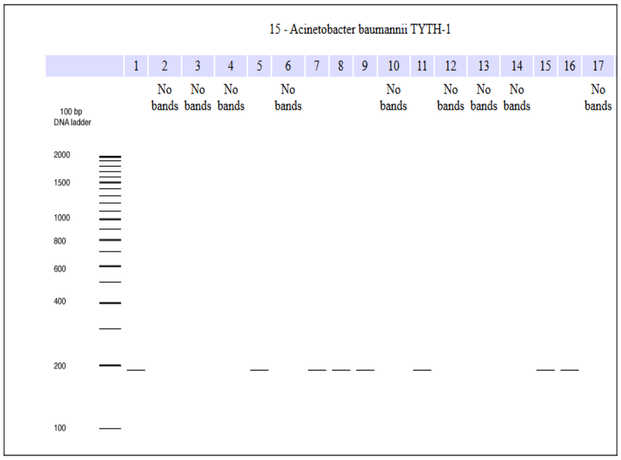

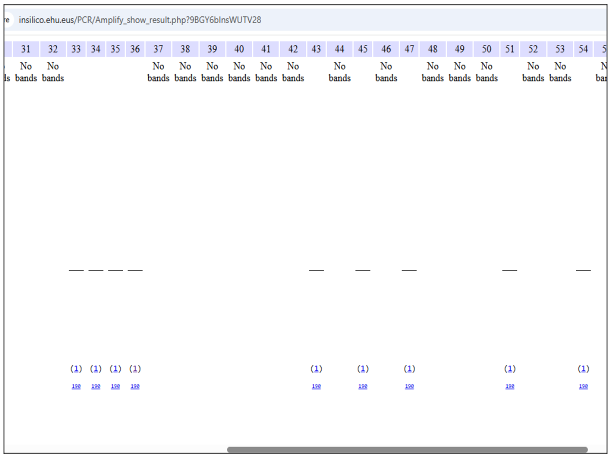

*Acinetobacter spp sul*2 products

Ecoli spp sul2 products

3A) *sul*3 primer set specificity evaluation using NCBI primer blast: (https://www.ncbi.nlm.nih.gov/tools/primer-blast

Overall: The newly designed *sul3* primer pair produced highly specific matches in the NCBI database at 248 bp, yielding 1,016 hits exclusively to *sul3* sequences across a wide range of bacterial species. These included *Raoultella ornithinolytica*, *Klebsiella spp.*, *Escherichia coli*, *Providencia stuartii*, *Salmonella* spp., *Proteus* spp., among others, indicating its potential utility as a universal *sul3* primer for direct PCR across diverse bacterial taxa.

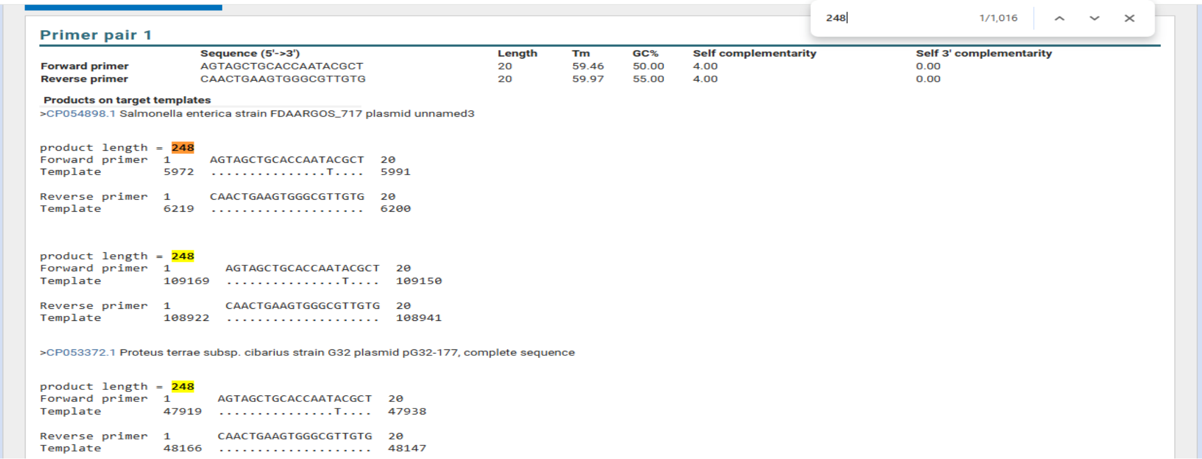

3B) *sul*3 primer set specificity evaluation using Insilco-PCR amplification: http://insilico.ehu.es/PCR/Amplify.php)

Overall, in-silico PCR (insilico.ehu.es) demonstrated that the *sul3* primer pair produced the expected 248-bp amplicon in multiple reference bacterial genomes, including*, Escherichia coli*, *Salmonella* spp., and *Klebsiella* spp., further supporting its specificity and suitability for broad detection of *sul3* across diverse taxa.

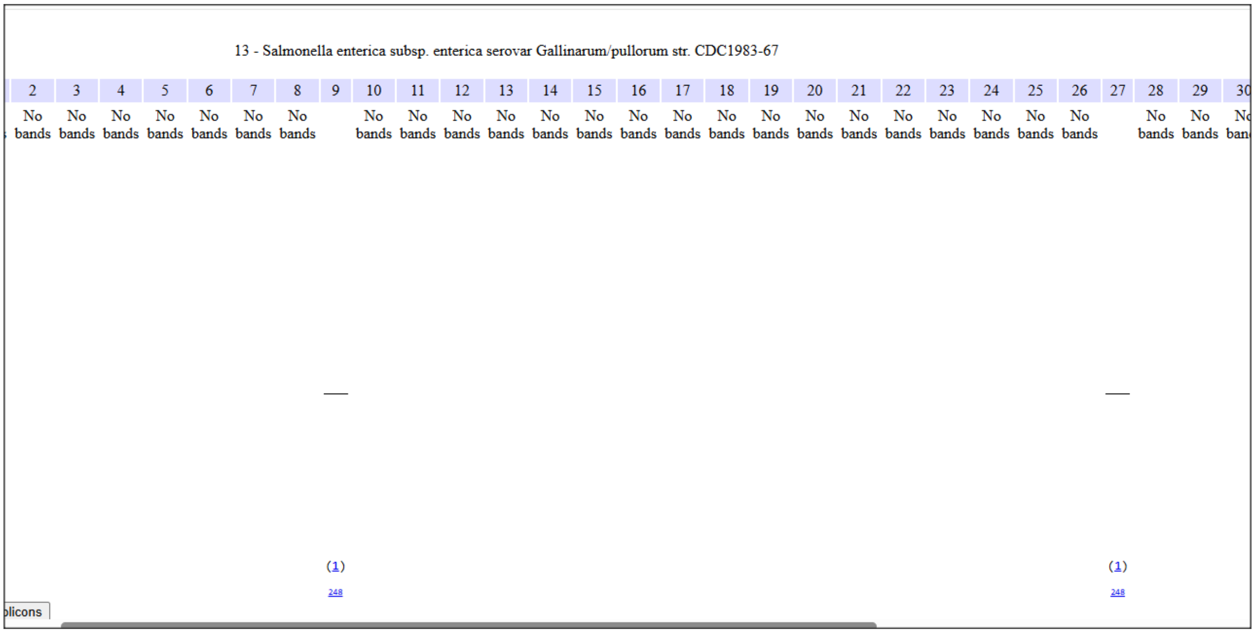

....

Part 2: PCR photos

1. ESBL Genes

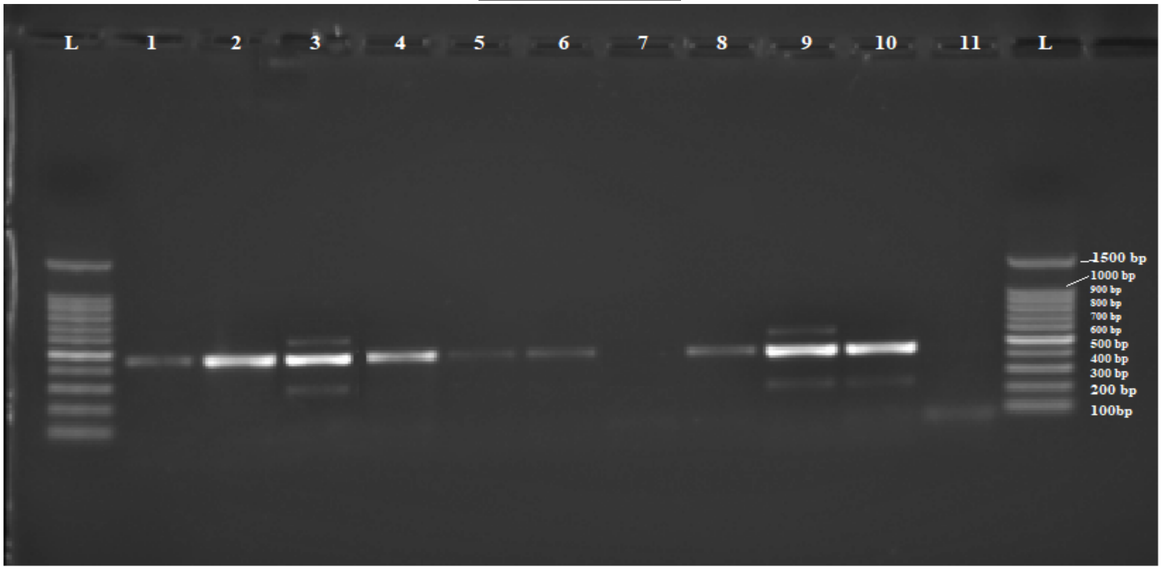

Figure 1: Multiplex PCR analysis of ESBL genes from Kalyoubia samples (Q1-Q10); Expected amplicon sizes: *bla*CTX-M (593 bp), ***bla*TEM (**445 bp) and ***bla*SHV** (237 bp); **L: Ladder (100 bp Biohelix); 3, 9: has all 3 genes *bla*CTX-M**, ***bla*TEM** and ***bla* SHV; 10: has both *bla*TEM** and ***bla*SHV**; 1, 2, 4, 5, 6, 8: has ***bla*TEM only; 7: is negative for all; 11: is negative control.**

…………………………………………

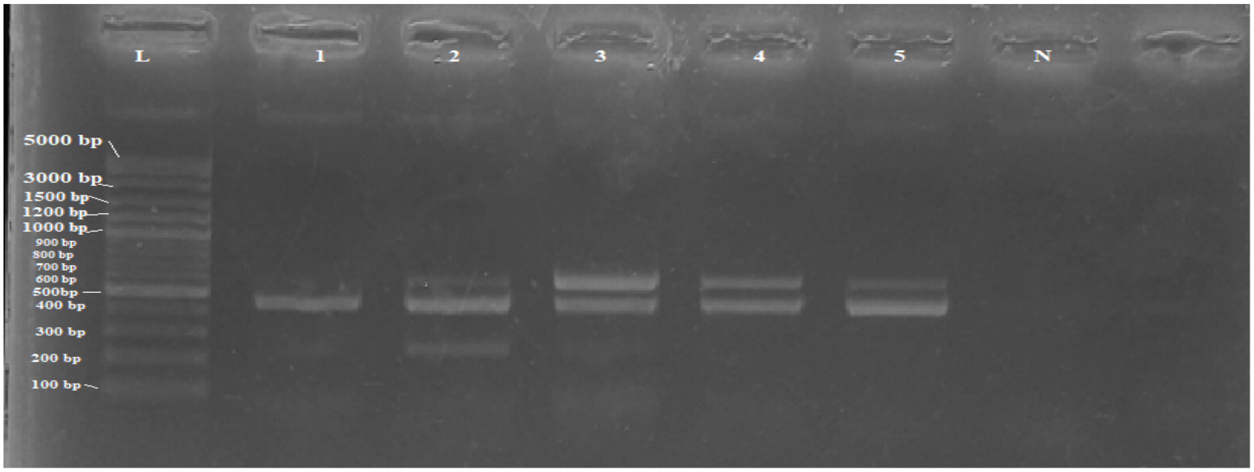

Figure 2: Multiplex PCR analysis of ESBL genes from Giza samples (G5-G10); Expected amplicon sizes: *bla*CTX-M (593 bp), ***bla*TEM (**445 bp) and ***bla*SHV (**237 bp); **L: Ladder (100 bp plus?); 2 : has all 3 genes *bla*CTX-M**, ***bla*TEM** and ***bla* SHV; 3, 4, 5 : has both *bla*TEM** and ***bla*CTX-M**; 1: has ***bla*TEM only; N: is negative control.**

……

2. <u>Carbapenem resistance Genes</u>

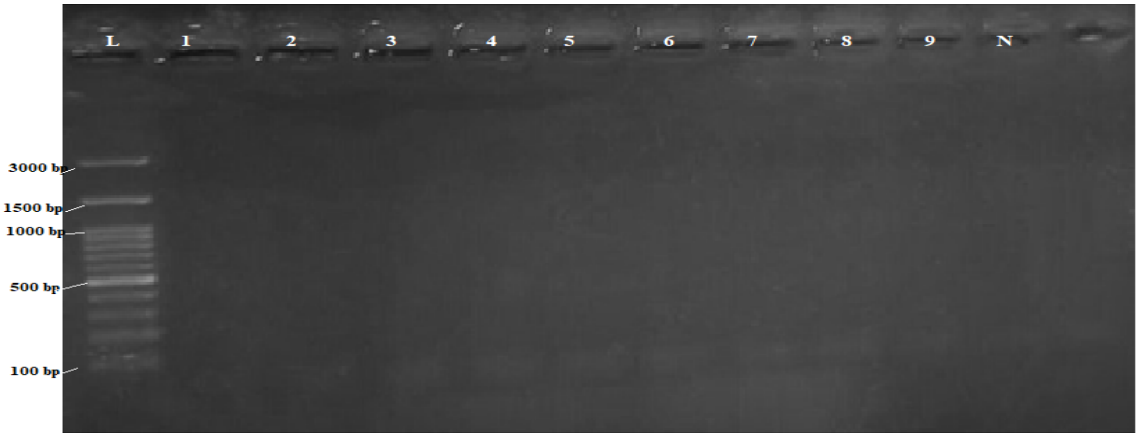

Figure 3: Multiplex PCR analysis of carbapenem resistance genes. Expected amplicon sizes blaOXA-48 (742 bp), ***blaNDM* (**621 bp), ***bla*KPC (**882 bp) and ***bla* VIM (**261 bp); **L: Ladder (100 bp H3 RTU HyLabs/ Genedirex); 1-9: were negative in all samples; N: is negative control.**

3. *bla*CMY-2 gene

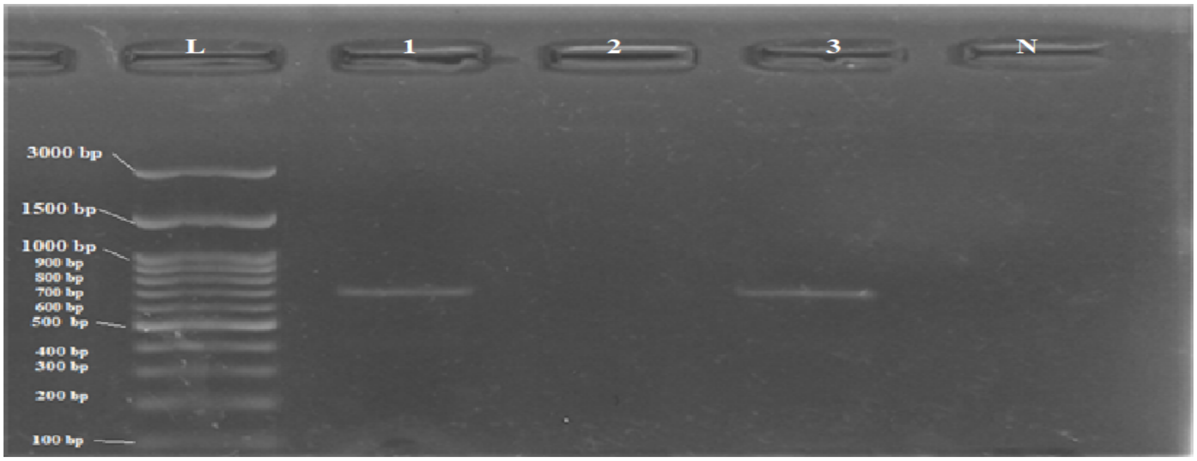

Figure 4: Uniplex PCR analysis of *bla*CMY-2 gene from Giza samples (G17-G21). Expected amplicon size *bla*CMY-2 gene (695 bp); **L: Ladder (100 bp H3 RTU HyLabs/ Genedirex); 1, 3: were positive; 2: was negative; N: is negative control.**

4. *tet*B & *mec*A

Only tetB

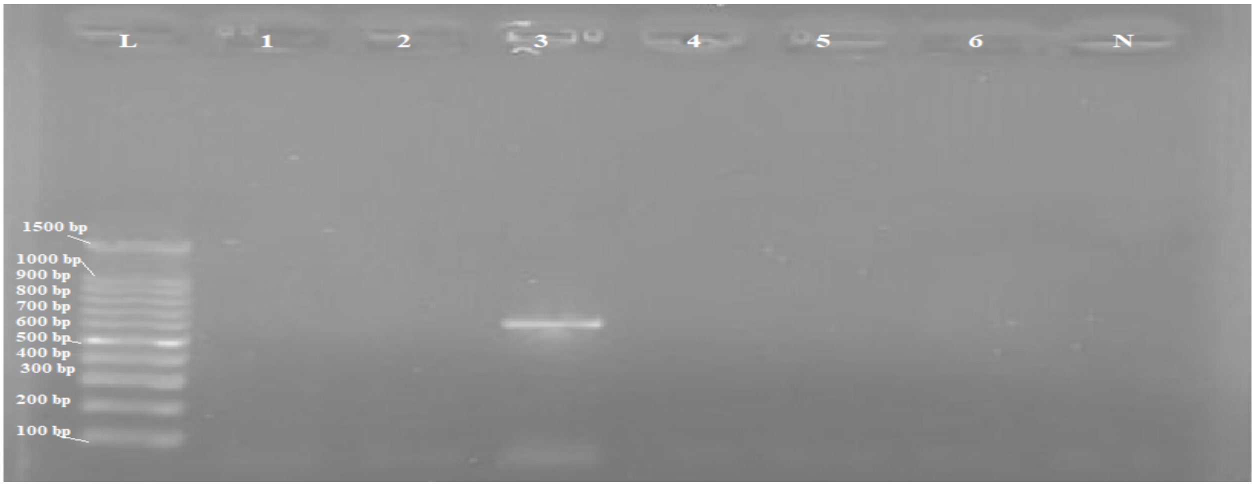

Figure 5: Duplex PCR analysis of tetB & mecA (G6). Expected amplicon sizes *mec*A (776b bp) and ***tet*B (**634 bp); **L: Ladder (100 bp, Biohelix); 3: has *tet*B only; 1, 2, 4, 5, 6: were negative for both genes; N: is negative control.**

………………………………………………….

Tet B and mecA Q9

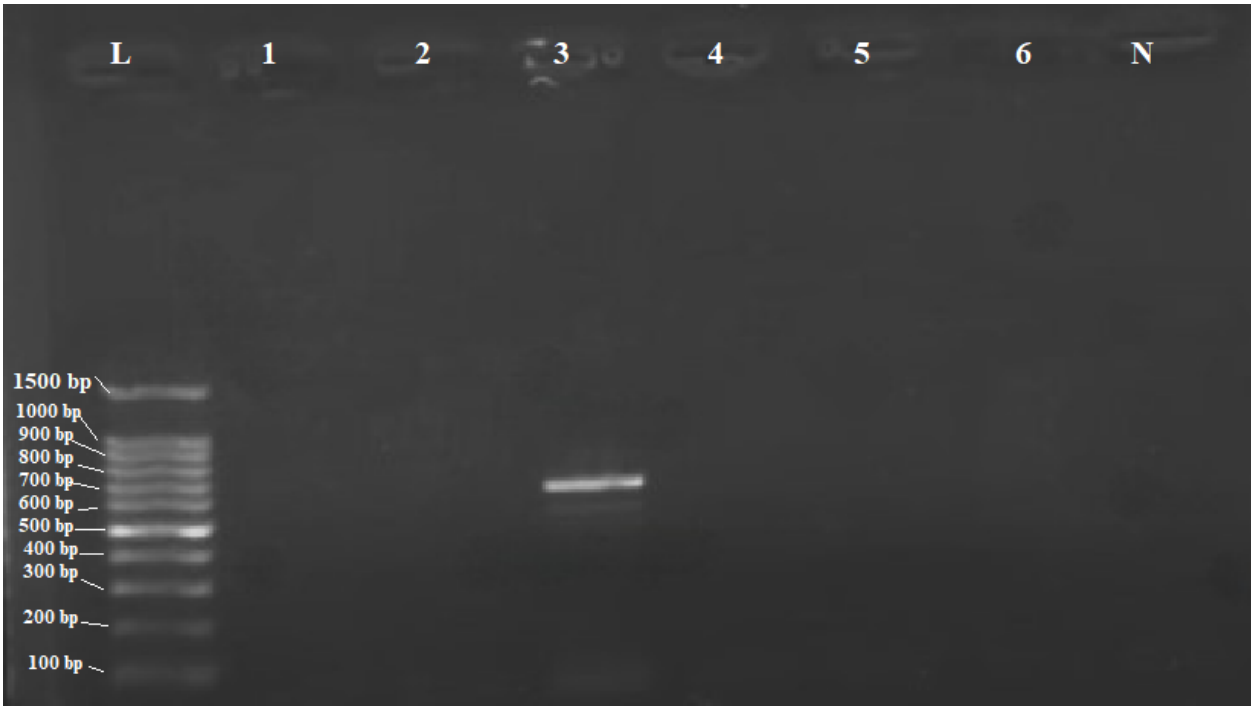

Figure 6: Duplex PCR analysis of tetB & mecA). Expected amplicon sizes *mec*A (776b bp) and ***tet*B (**634 bp); **L: Ladder (100 bp, Biohelix); 3: has Both *tet*B and *mec*A; 1, 2, 4, 5, 6: were negative for both genes; N: is negative control.**

………

5. <u>tetA and sulA</u>

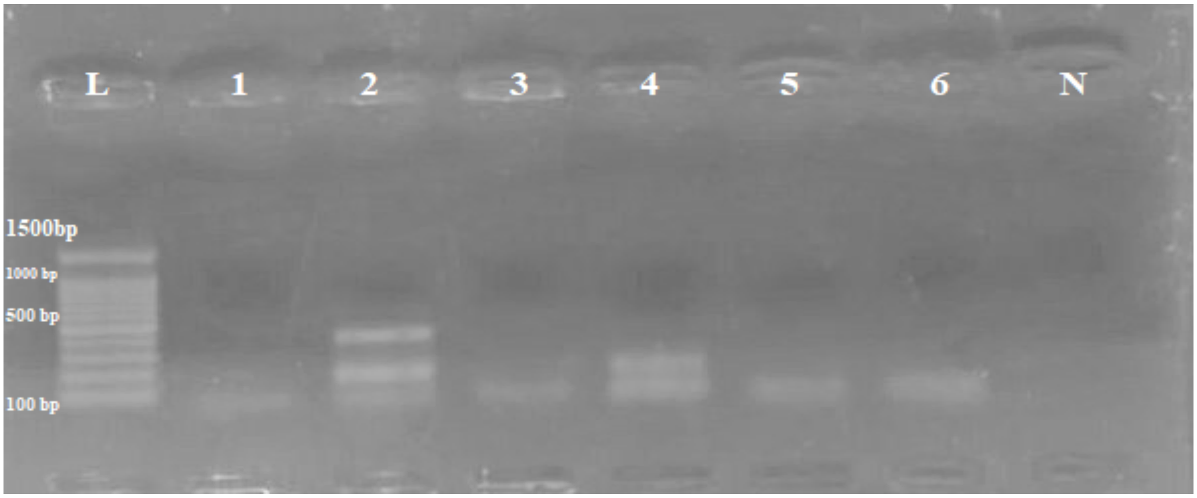

Figure 7: Duplex PCR analysis of *tet*A & *sul*1 from Kalyoubia samples (Q1-Q10). Expected amplicon sizes *tet*A (372 bp) and *sul1* (162 bp); L: Ladder (100 bp, Biohelix); 2: has Both *tetA* and *sul1*; 2: has is positive for both genes; 4: has *sul*1 gene only; 1, 3, 5, 6: negative for both genes; N: is negative control.

……………………………………….

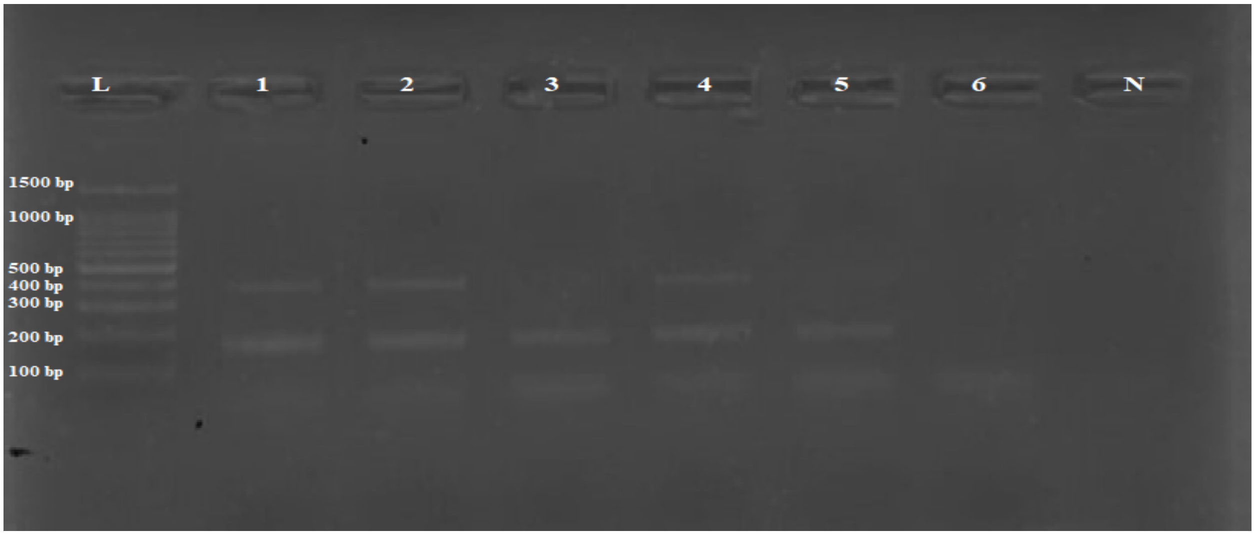

Figure 8: Duplex PCR analysis of *tet*A & *sul*1 from Kalyoubia samples (Q11-Q16). Expected amplicon sizes *tet*A (372 bp) and *sul1* (162 bp); L: Ladder (100 bp, Biohelix); 1, 2 ,3 : has Both *tetA* and *sul1*; 3, 5: has *sul*1 gene only; 6: negative for both genes; N: is negative control.

…………………………….

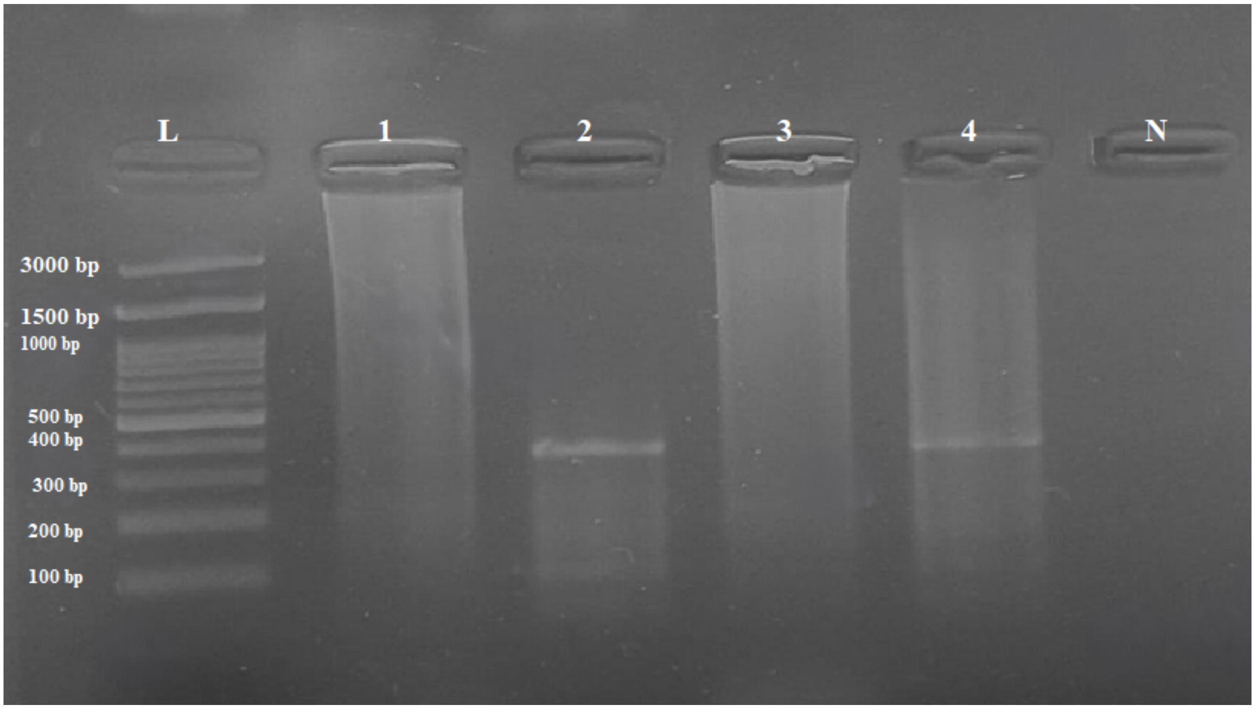

TetA only G5/G19/G20

Figure 9: Duplex PCR analysis of *tet*A & *sul*1 from Giza samples (G5). Expected amplicon sizes *tet*A (372 bp) and ***sul1* (**162 bp); **L: Ladder (100 bp, H3 RTU HyLabs/ Genedirex); 2 ,4 : has *tetA gene* only; 1, 3: negative for both genes; N: is negative control.**

....

6. *tet*M, *par*C & *van*B

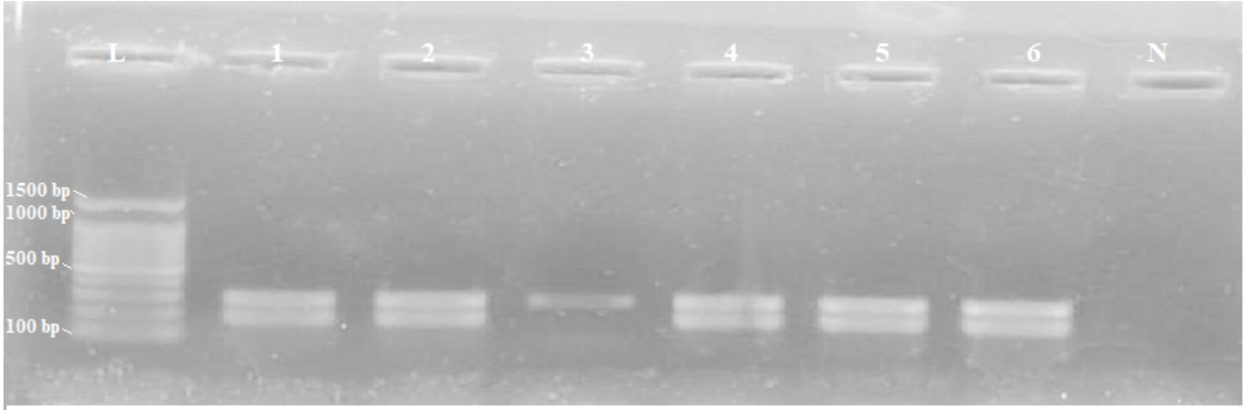

Figure 10: Multiplex PCR analysis of *tet*M, *par*C, *van*B genes from Kalyoubia samples (Q11-Q16); Expected amplicon sizes: *vanB* (536bp), ***parC* (**287bp) and ***tetM***

(171 bp); **L: Ladder (100 bp Biohelix); 1, 2, 4, 5, 6: has both *tetM and parC*** genes; 3: has ***parC*** **only;N: is negative control.**

……………………………………………

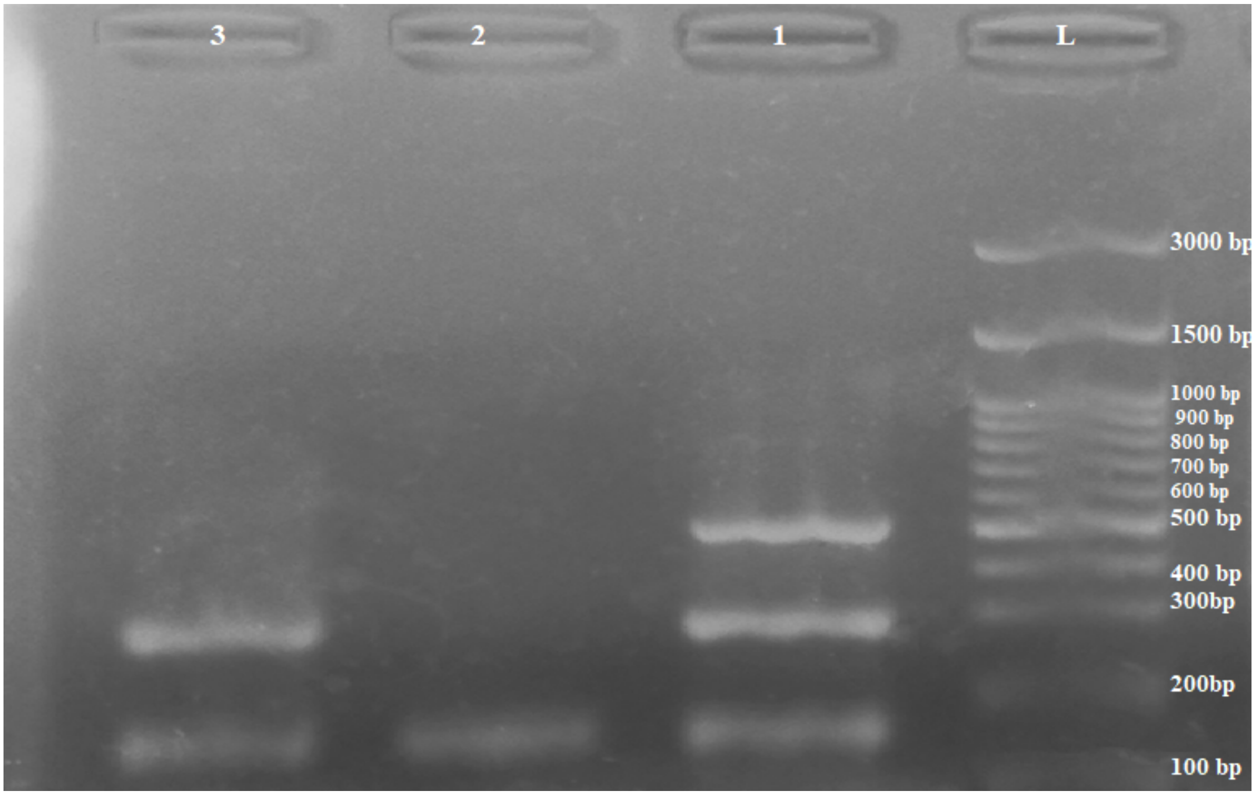

Figure 11: Multiplex PCR analysis of *tet*M, *par*C, *van*B genes from Giza samples (G16-Q19); Expected amplicon sizes: *vanB* (536bp), ***parC*** (287bp) and ***tetM* (**171 bp); **L: Ladder (100 bp , H3 RTU HyLabs/ Genedirex); 1: has 3 genes, *van*B, *tetM and parC*** genes; 2: has ***tetM* only; 3: has both *parC*** and ***tetM*; N: is negative control.**

……………………….

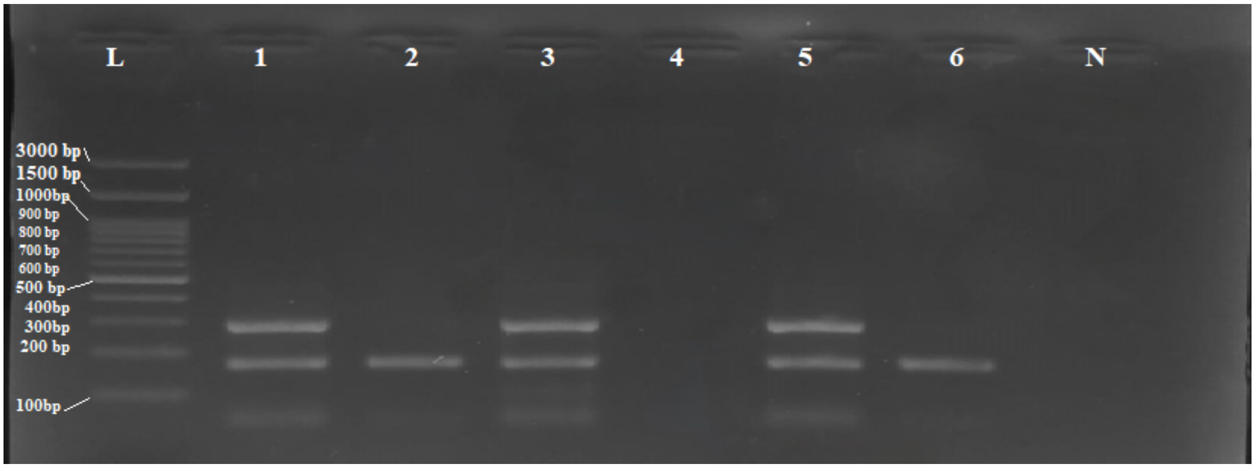

Figure 12: Multiplex PCR analysis of *tet*M, *par*C, *van*B genes from Kalyoubia samples (Q1-Q10); Expected amplicon sizes: *vanB* (536bp), ***parC* (**287bp) and ***tetM* (**171 bp); **L: Ladder (100 bp H3 RTU HyLabs/ Genedirex); 1, 3, 5: has all 3 genes: *vanB, tetM and parC*** genes; 2,6: has ***tetM* only;4: is negative; N: is negative control.**

....

7. <u>Sul2 genes</u>

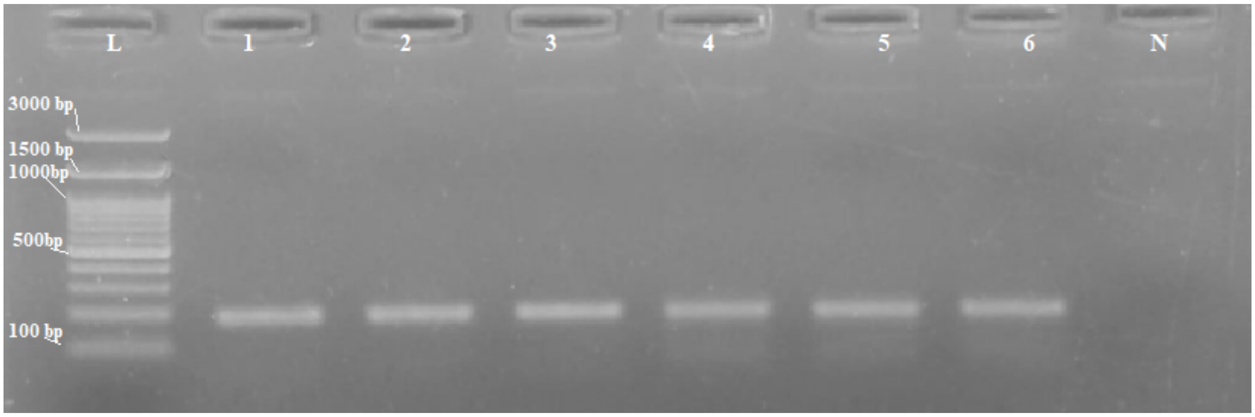

Figure 13: Uniplex PCR analysis of *sul*2 gene from Giza samples (G5-G10). Expected amplicon size *sul*2 gene (190 bp); **L: Ladder (100 bp H3 RTU HyLabs/ Genedirex); 1, 2, 3, 4, 5, 6: were positive; N: is negative control.**

....

....

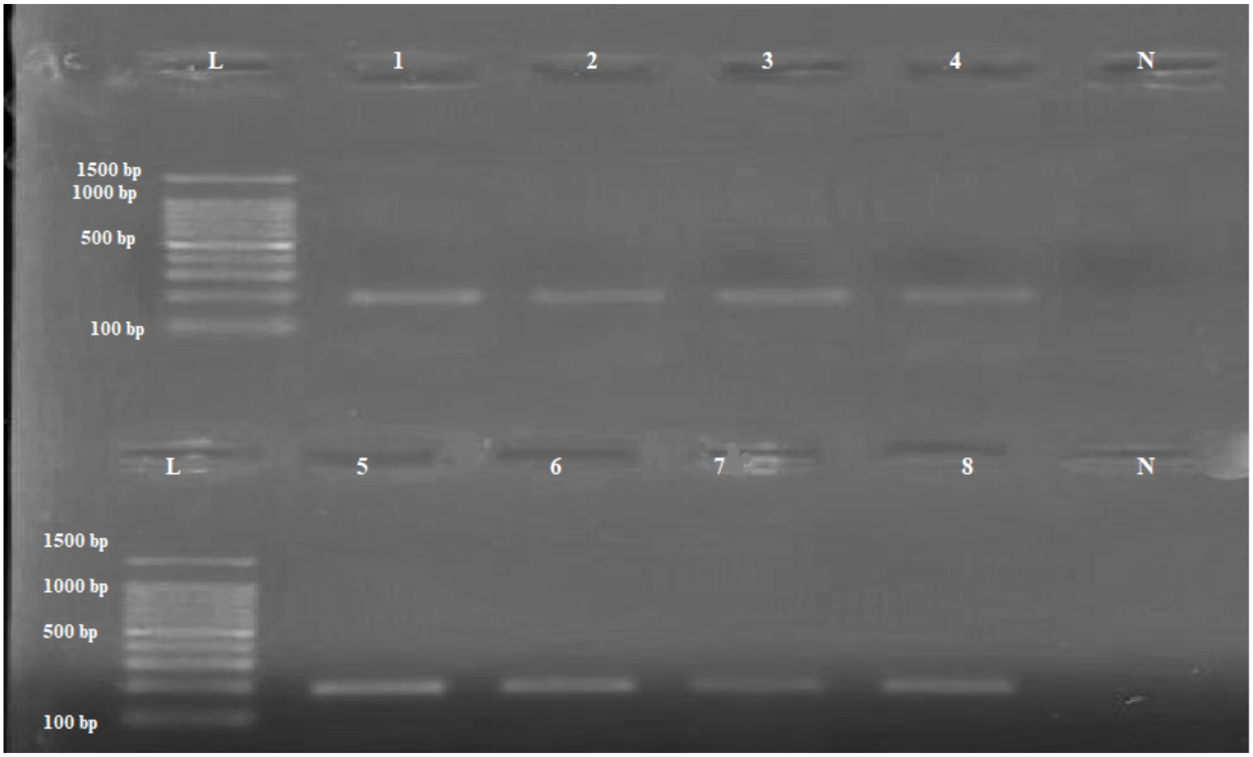

Figure 14: Uniplex PCR analysis of *sul*2 gene from Giza (G1-G4) and Kalyoubia samples (Q20-Q24). Expected amplicon size *sul*2 gene (190 bp); **L: Ladder (100 bp Biohelix)); 1, 2, 3, 4, 5, 6, 7, 8: were positive; N: is negative control.**

....

8. <u>Sul3 genes</u>

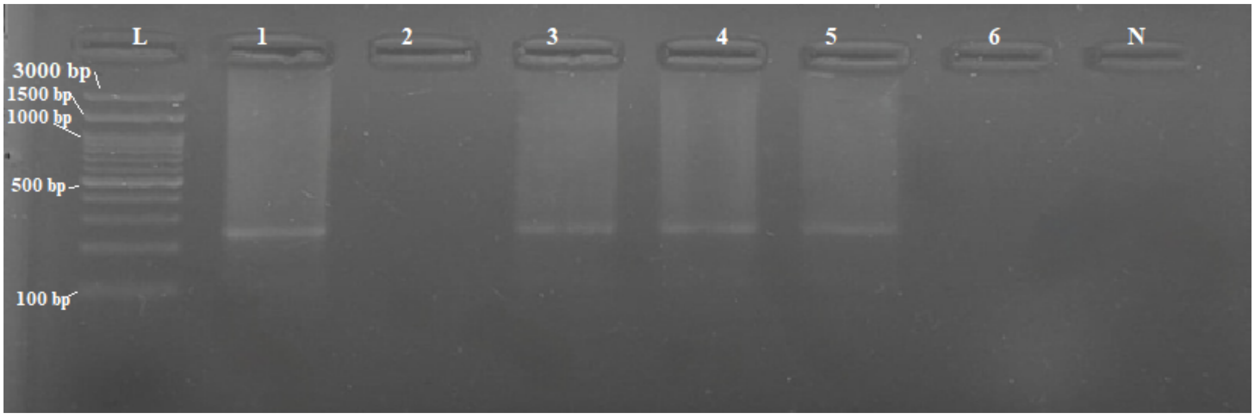

Figure 15: Uniplex PCR analysis of *sul*3 gene from Kalyoubia samples (Q10-Q15). Expected amplicon size *sul*3 gene (248 bp); **L: Ladder (100 bp H3 RTU HyLabs/ Genedirex)); 1, 3, 4, 5: were positive; 2, 6: were negative; N: is negative control.**

……………………………

………

9. qnrA gene

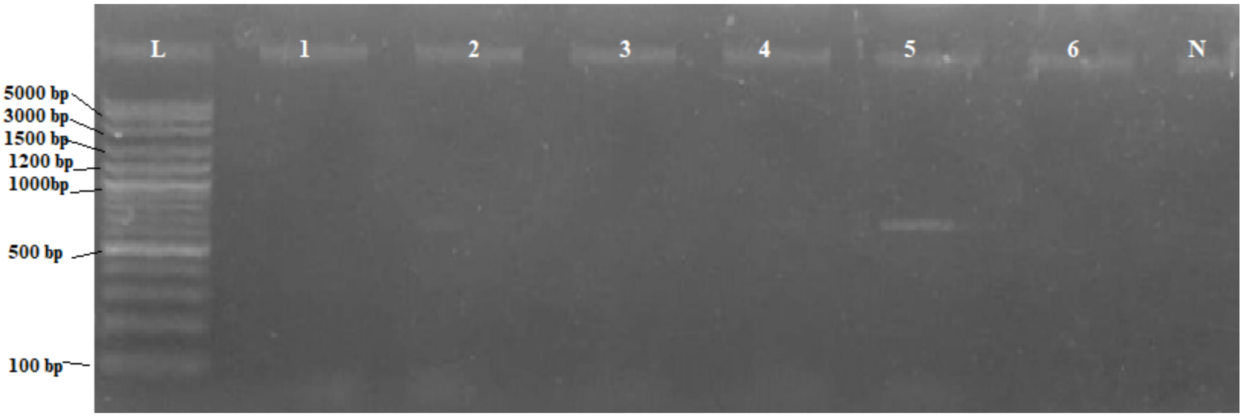

Figure 16: Uniplex PCR analysis of *qnr*A gene from Kalyoubia samples (Q11-Q16). Expected amplicon size *qnr*A gene (661 bp); **L: Ladder (100 bp?); 5: were positive; 1, 2, 3, 4, 6: were negative; N: is negative control.**

………

10. qnrB gene

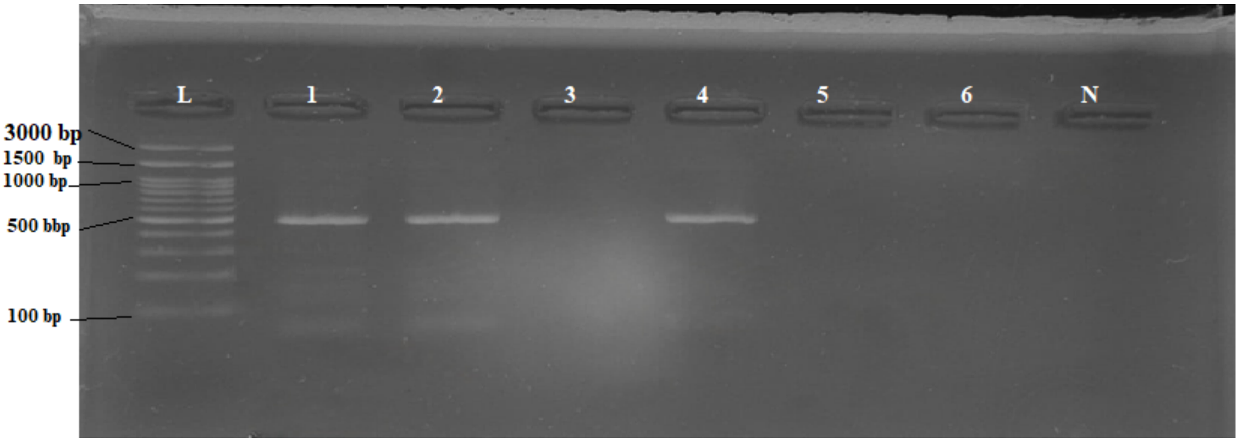

Figure 17: Uniplex PCR analysis of *qnr*B gene from Giza samples (G1, G2, G11). Expected amplicon size *qnr*B gene (495 1 bp); **L: Ladder (100 bp H3 RTU HyLabs/ Genedirex); 1, 2, 4: were positive; 3, 5, 6: were negative; N: is negative control.**

....

11. Qnrs Gene

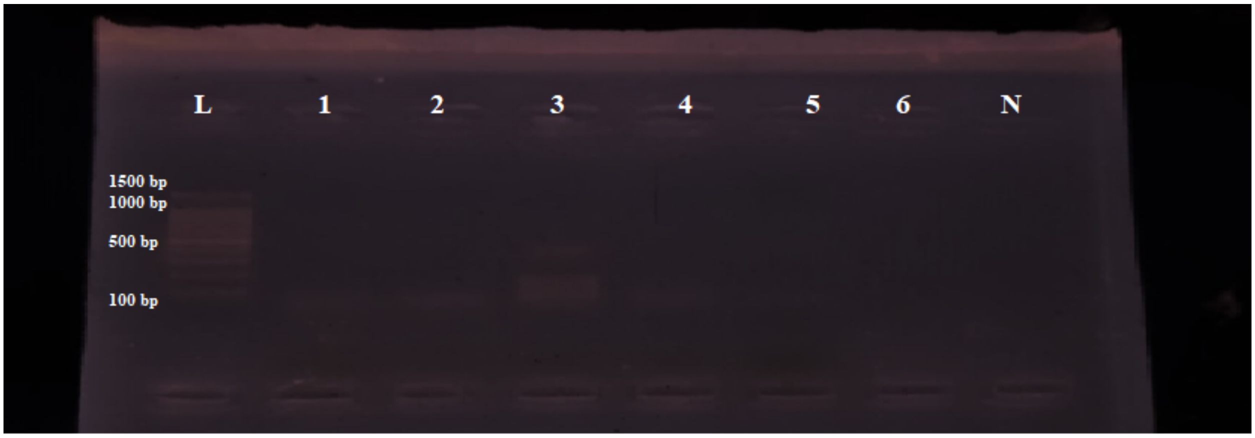

Figure 18: Uniplex PCR analysis of *qnr*S gene from Kalyoubia samples (Q9). Expected amplicon size *qnr*B gene (118 bp); **L: Ladder (100 bp Biohelix); 3: was positive; 1, 2, 4, 5, 6: were negative; N: is negative control.**

....

....

12. ArmA gene

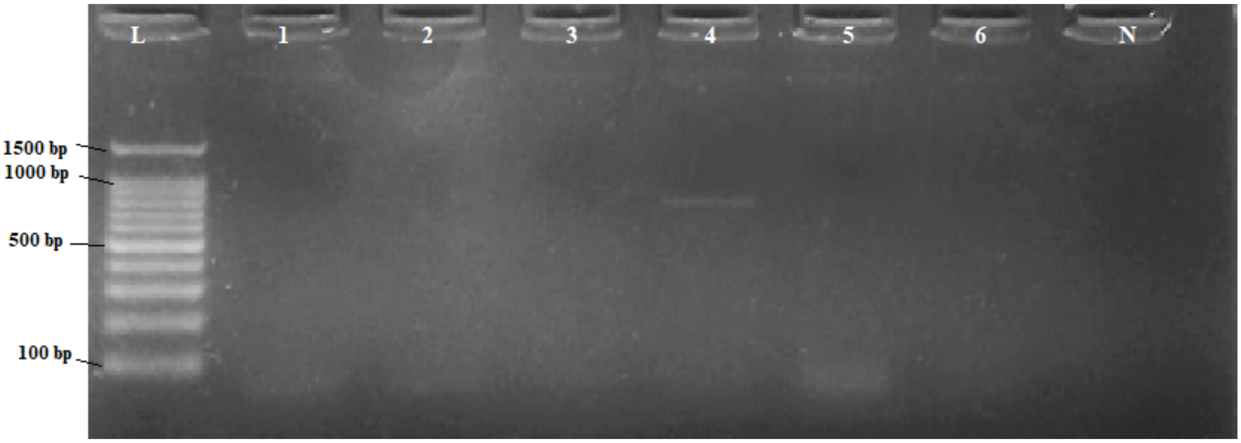

Figure 19: Uniplex PCR analysis of *arm*A gene from Kalyoubia samples (Q3-10). Expected amplicon size *arm*A gene (776 bp); L: Ladder (100 bp Biohelix); 4: was positive; 1, 2, 3, 5, 6: were negative; N: is negative control.

ARMA Q3-Q10

………………………………………

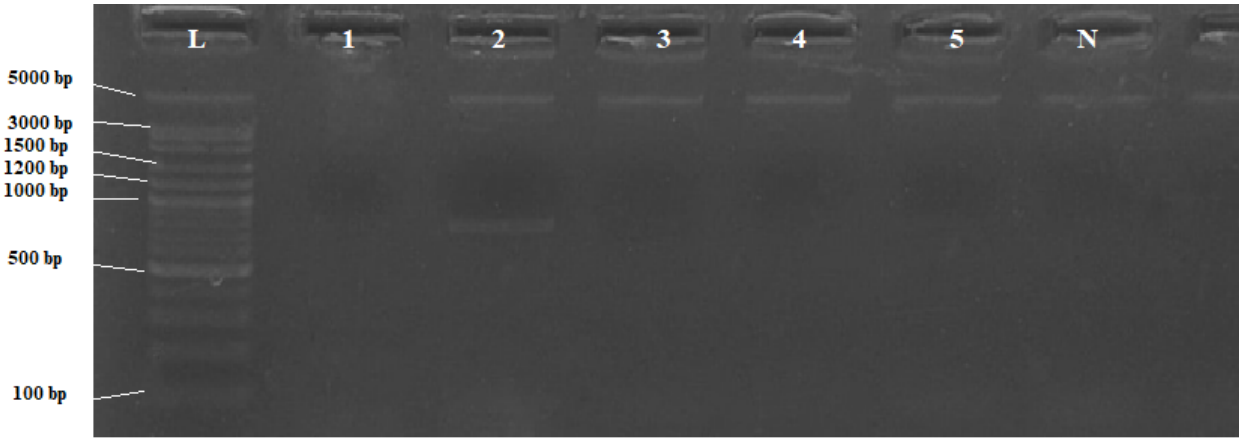

Figure 20: Uniplex PCR analysis of *arm*A gene from Kalyoubia samples (Q11-16). Expected amplicon size *arm*A gene (776 bp**); L: Ladder (100 bp?); 2: was positive; 1, 3, 4, 5, : were negative; N: is negative control.**

……

13.<u>Aac 3 and int1 Gene</u>

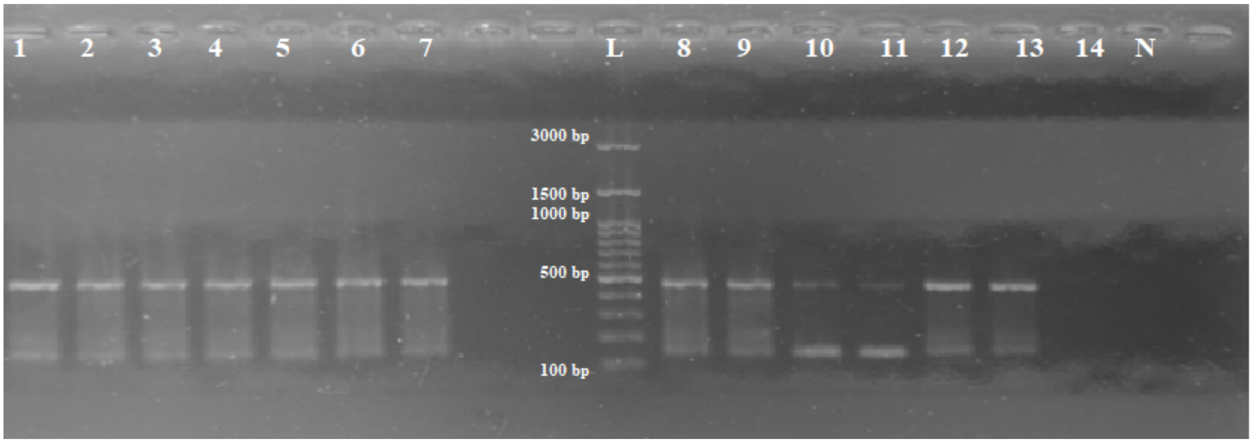

Figure 20: Uniplex PCR analysis of *aac3-la* gene from Kalyoubia samples (Q1-10). Expected amplicon size *aac*3-la gene (484 bp) and *intl* (146 bp); **L: Ladder (100 bp H3 RTU HyLabs/ Genedirex); 1, 2, 3, 4, 5, 6, 7, 8, 9, 10, 11, 12, 13: was positive both *aac*3-la gene** and *intl*1**;14: were negative; N: is negative control.**

……………………………………………………………………………………….

Aac 3 and int1 Gene

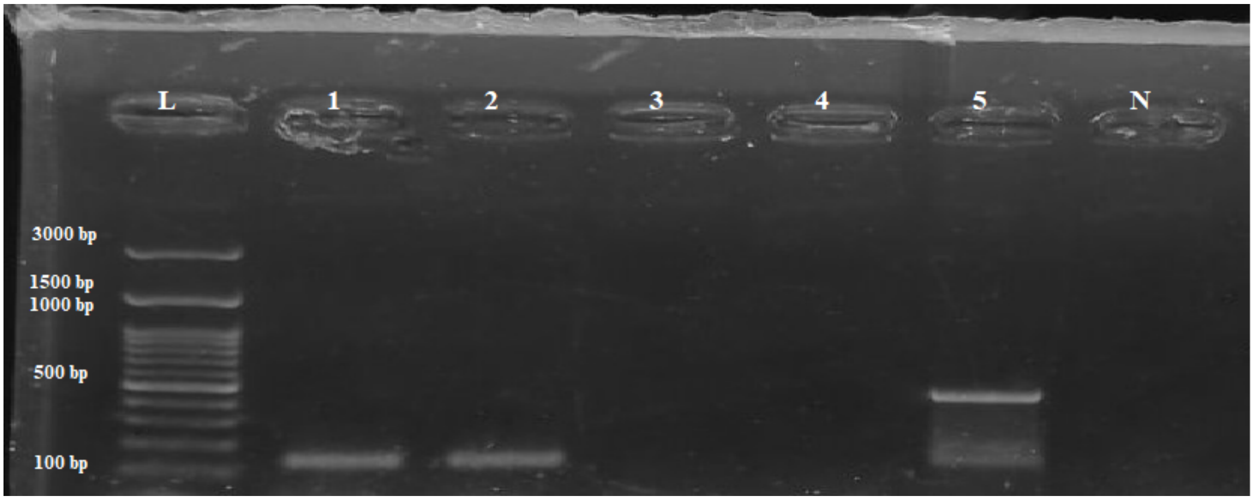

Figure 21: Uniplex PCR analysis of *aac3-la* gene from Giza samples (G1-5). Expected amplicon size *aac*3-la gene (484 bp) and *intl* (146 bp); **L: Ladder (100 bp H3 RTU HyLabs/ Genedirex); 5 : was positive both *aac*3-la gene** and *intl*1; **1, 2: were positive *int*1 only; 3, 4: were negative both genes; N: is negative control.**

AAC+INT G1-G5)
