# Supplementary material for: Antimicrobial resistance profiling in poultry industry: a culture-independent resistome analysis and risk factor assessment
Source: BMC Vet Res. 2026 Mar 14;22:212. doi: 10.1186/s12917-026-05334-w (PMC13063522; doi:10.1186/s12917-026-05334-w)
Supplement: Supplementary file 3 — Supplementary Material 3. [file 12917_2026_5334_MOESM3_ESM.pdf]

Antimicrobial Resistance Profiling In Poultry Industry: A Culture-Independent Resistome Analysis and Risk Factor Assessment

Sabah Ali[a*], Mariam Hassan[b,c*], Tamer Essam[b], Shaymaa Abdelmalek Mohamed[a], Khaled F. Al-Amry[a]

[a]*Department of Microbiology, Faculty of Veterinary Medicine, Cairo University, Giza, Egypt.*

[b]*Department of Microbiology and Immunology, Faculty of Pharmacy, Cairo University, Cairo, Egypt.*

[c]*Department of Microbiology and Immunology, Faculty of Pharmacy, Galala University, New Galala City, Suez, Egypt*

Supplementary file S1

AMR Questionnaire Survey

(Sampling Data Sheet)

(English format)

Name :-

Contact information:-

**General sampling information :**
1. **Sampling district/governorate?**<br><br>----------------------- --------------------------------
2. **Sample code?**<br><br>------------------------------
3. **Date (season)?**<br><br>-----------------------------
4. **Number of samples?**<br><br>-----------------------------
5. **Type of samples?**<br><br>○ cloacal swaps

○ organs
○ litter
○ Other

6. **Collection site?**

○ Pen
○ Poultry inspection lab
○ Slaughter house
○ Other

Poultry demographic data :

7. **Type of poultry farm?**

○ Breeders
○ Layers
○ Broilers

8. **What is the breed of sampled chickens?**

----

9. **What is the age of sampled chickens? (…………….)**

○ 2 weeks to 1month
○ > 1 month to 2 months
○ > 2 month

10. **What is the health status of sampled chickens?**

○ Apparently Healthy
○ Sick
○ Dead

Bird's case history :

11. **What is the suspected diagnosis for these chickens, based on the vet's assessment?**

------------ ------------ ---------------- --------------------- - -----------------------------
----------- --------------------- ----------------------- --------------------------------

12. **What is the current average mortality rate in your farm?**

----

farm characteristics :

13. **What is the size of the chicken flock in your farm?**

**14. What kind of poultry housing system is used for these birds?**<br><br>○ Cages<br>○ Floor<br>○ Other		
**Biosafety measures (10points) :**		
**15.** Are vaccination programs used?	Yes ☐ Details: …………<br>…………………………..<br>….. ……………….....	No ☐
**16.** Are sick animals quarantined in separate areas?	Yes ☐	No ☐
**17.** Does cleaning and disinfection of equipment and utensils occur regularly?	Yes ☐	No ☐
**18.** Are foot dips and wheel baths present at the entry of farm?	Yes ☐	No ☐
**19.** Is a fence present around premises & far away from adjacent farms?	Yes ☐	No ☐
**20.** Does farm have proper ventilation?	Yes ☐	No ☐
**21.** Are there any insects, pets or rodents present on farm premises?	Absent ☐	Present ☐
**22.** Is there a restriction on visitors entering the poultry area?	Yes ☐	No ☐
**23.** Are antibiotics given through prescription by a veterinarian?	Yes ☐	No ☐
**24.** Are carcasses and litter removed properly (burial/burned)?	yes ☐	No ☐
Biosecurity points	High (……) ☐	low (….) ☐
**Data on antibiotics used:**		
**25. What are the names (commercial/generic) of the antibiotics given to flock? With or without prescribtion.?**<br><br>**---------------- ----------------------- ---------------- --------------------------------**<br>**---------------- -------------------------------- ---------------------------------------**		
**26. Are there other medications used? With example?**<br><br>……………………… …………………………………………… ………………<br>…………………………………………….. …………………………………….		
